# Whole metagenome sequencing and 16S rRNA gene amplicon analyses reveal the complex microbiome responsible for the success of enhanced in-situ reductive dechlorination (ERD) of a tetrachloroethene-contaminated Superfund site

**DOI:** 10.1371/journal.pone.0306503

**Published:** 2025-02-14

**Authors:** Rebecca A. Reiss, Peter A. Guerra, Oleg Makhnin, Matthew Kellom

**Affiliations:** 1 Biology Department, New Mexico Tech, Socorro, New Mexico, United States of America; 2 LifeScience Testing and Analysis, Albuquerque, New Mexico, United States of America; 3 Lynker Corporation, Albuquerque, New Mexico, United States of America; 4 Mathematics Department, New Mexico Tech, Socorro, New Mexico, United States of America; 5 Department of Energy Joint Genome Institute, Lawrence Berkeley National Laboratory, Berkeley, California, United States of America; Universidade Estadual de Ponta Grossa, BRAZIL

## Abstract

The North Railroad Avenue Plume (NRAP) Superfund site in New Mexico, USA exemplifies successful chlorinated solvent bioremediation. NRAP was the result of leakage from a dry-cleaning that operated for 37 years. The presence of tetrachloroethene biodegradation byproducts, organohalide respiring genera (OHRG), and reductive dehalogenase (*rdh*) genes detected in groundwater samples indicated that enhanced reductive dechlorination (ERD) was the remedy of choice. This was achieved through biostimulation by mixing emulsified vegetable oil into the contaminated aquifer. This report combines metagenomic techniques with site monitoring metadata to reveal new details of ERD. DNA extracts from groundwater samples collected prior to and at four, 23 and 39 months after remedy implementation were subjected to whole metagenome sequencing (WMS) and 16S rRNA gene amplicon (16S) analyses. The response of the indigenous NRAP microbiome to ERD protocols is consistent with results obtained from microcosms, dechlorinating consortia, and observations at other contaminated sites. WMS detects three times as many phyla and six times as many genera as 16S. Both techniques reveal abundance changes in *Dehalococcoides* and *Dehalobacter* that reflect organohalide form and availability. Methane was not detected before biostimulation but appeared afterwards, corresponding to an increase in methanogenic *Archaea*. Assembly of WMS reads produced scaffolds containing *rdh* genes from *Dehalococcoides*, *Dehalobacter*, *Dehalogenimonas*, *Desulfocarbo*, and *Desulfobacula*. Anaerobic and aerobic cometabolic organohalide degrading microbes that increase in abundance include methanogenic *Archaea*, methanotrophs, *Dechloromonas*, and *Xanthobacter*, some of which contain hydrolytic dehalogenase genes. Aerobic cometabolism may be supported by oxygen gradients existing in aquifer microenvironments or by microbes that produce O_2_ via microbial dismutation. The NRAP model for successful ERD is consistent with the established pathway and identifies new taxa and processes that support this syntrophic process. This project explores the potential of metagenomic tools (MGT) as the next advancement in bioremediation.

## Introduction

### Tetrachloroethene prevalence and microbial biodegradation

The use of tetrachloroethene (PCE) for dry cleaning began in the 1930s but health concerns were not recognized until the 1960s [1]. Contamination of groundwater with PCE from its use in dry-cleaning and in degreasing threatens potable water supplies worldwide [2]. Complete microbial anaerobic respiration of PCE consists of a sequential process in which successive reductase enzymes remove chlorine atoms (Cl) to ultimately yield ethene, which is readily mineralized by soil microbes and is not known to be a carcinogen. Chlorinated volatile organic compounds (cVOCs) such as PCE and its degradation byproducts vary in toxicity [3]. PCE is a probable carcinogen (group 2A) for all routes of exposure: inhalation, ingestion, or dermal. PCE reductase enzymes cleave the first Cl, generating trichloroethene (TCE), a carcinogen (group 1) regardless of exposure route. Dichloroethene (DCE, cis and/or trans isomers), vinyl chloride (VC), and ethene are formed in succession. There is inadequate evidence to assign a carcinogenicity level to the isomers of 1,2 DCE [4, 5], but exposure can cause central nervous system depression, liver damage, and infertility. Although 1,1 DCE is classified as a possible human carcinogen [6], it is not a product of ERD and was not detected at NRAP. VC is carcinogenic (group 1) by any route of exposure. During each step in the reduction byproduct solubilities increase and their adsorption to soil decrease, increasing their mobility and concentration in groundwater. Complete reduction to ethene without stalling is the goal of ERD, otherwise bioremediation could result in an increased exposure to toxic byproducts.

Organohalides (OH), including VC, are produced during natural biogeochemical processes and microbial transformations of chlorinated hydrocarbons contribute to the global Cl cycle [7–9]. Dehalogenation mechanisms are widespread in nature and account for the detection of taxa capable of respiring OH at sites undergoing natural attenuation [10, 11]. Enhanced in-situ reductive dechlorination (ERD) protocols adjust groundwater to anaerobic reduced conditions, which establishes a favorable environment for microbes containing reductive dehalogenase (*rdh)* genes to respire chlorinated solvents. There are currently 30 bacterial genera validated in the literature that include strains capable of dechlorinating ethenes through anaerobic respiration (S2 Table in S1 File). Collectively, *rdh* gene products regardless of the substrate used, make up the protein family (pfam) 13486, based on the alignment of amino acids common to proteins with reductive dehalogenase function [12]. The relationship of OH-respiring genera *Dehalococcoides* (*Dhc*) and *Dehalobacter* (*Dhb)* is well-established, *Dhb* dominates during the presence of PCE, TCE, and DCE (also chlorinated ethanes), *Dhc* increases as VC becomes available [13]. ERD is an example of ‘obligately mutualistic metabolism,’ a form of syntrophy that entails the action of multiple microbes to make an energetically unfeasible metabolic process attainable [14]. Syntrophy includes the importance of electron transfer and provides a conceptual framework to understand ERD from the community ecology perspective.

*Geobacter* plays a complex role in chloroethene biodegradation. This genus is known for the transfer of electrons to other microbes along specialized pili that can conduct electrons, known as nanowires [15]. *Geobacter lovleyi* and *G*. *thiogenes* are distinguished by the presence of *rdh* genes in these genomes and both are assigned to the genus *Trichlorobacter* in Joint Genome Institute’s (JGI) Integrated Microbial Genome/Microbiomes (IMG/M) database [16]. This is based on the legitimate and validly published proposal to establish a new genus [17]. *Geobacter* produces cobalamin that can be transferred to *Dhc*, a cobalamin auxotroph [18]. *Sulfurospirillum*, *Acetobacterium* and *Desulfitobacterium* are also cobalamin producers found in dechlorinating consortia [19, 20]. The dependence of *Dhc* on such corrinoid-producing microbes to supply this critical cofactor for OH respiration represents the importance of syntropy during ERD [14].

Dechlorination that occurs through pathways other than respiration is referred to as cometabolism [21]. The list of genera capable of cometabolizing OH (S3 Table in S1 File) includes methanogenic *Archaea*, first observed in association with dechlorinating communities in the 1980s [22]. In the 1990s it was reported that methanogens harbor reduced transition-metal cofactors capable of dechlorinating TCE in anaerobic conditions [23, 24]. Meta-analysis of OH contaminated sites confirms the linkage between reductive dechlorination and methanogenic taxa [24].

Once PCE is reduced to TCE, it can be a substrate for oxidation by methane and toluene monooxygenases (*mmo* and *tmo*, collectively *mo*), harbored by methanotrophs, forming unstable epoxides [25–27]. Epoxide detection is difficult and the fate of these molecules is unclear. If converted into chlorinated ethanes, these can be respired by strains of *Dhb*, *Trichlorobacter*, and *Desulfitobacterium* [28–30]. Hydrolytic dehalogenase genes (*hdh*) are diverse, widespread in nature, and harbor the potential to complete dechlorination of OHs [10].

Although the anaerobic conditions for ERD should exclude aerobic cometabolism, oxygen gradients may exist in microenvironments at the aquifer soil interface [21]. Another possibility is the presence of microbes capable of producing oxygen without photosynthesis through chlorite or nitric oxide dismutation [31]. Dark oxygen is formed by chlorite dismutase (Cld, EC.1.13.11.49, a.k.a. chlorite O2-lyase), producing Cl^-^ and O_2_, and nitric oxide dismutase (Nod, EC.14.12.17, a.k.a. nitric oxide dioxygenase), which releases N_2_ and O_2_. The presence of *cld* and *nod* genes in subsurface microbial communities can explain the presence of molecular oxygen that supports aerobic metabolism in an anaerobic environment [31].

The term microbiome is commonly used to refer to bacterial communities associated with a host, such as the human gut microbiome. The same metagenomic techniques can be applied to microbiomes associated with abiotic environments, known as environmental microbiomes (e-biomes) and includes microbes associated with soil, water, air, and built environments. Examples of built environments are homes, public transportation, drinking water and sewer utilities, food processing plants, and facilities constructed to remediate contaminated environments.

### History of the North Railroad Avenue Plume (NRAP)

NRAP was the result of leakage of PCE from the Norge Town Laundry and Dry Cleaners located on North Railroad Avenue into the sole-source potable water aquifer for the city of Española New Mexico, the Santa Clara Pueblo, and nearby rural populations. The facility operated from 1970 to 2007, resulting in a toxic plume that extended approximately 1.2 km from the source and reached a depth of about 80 m below ground surface (bgs). PCE, TCE, and DCE commingled with petroleum hydrocarbon contamination in the plume, which was discovered in 1989 and was declared a United States Environmental Protection Agency (EPA) Superfund Site in 1999 (National Priority List #NMD986670156). NRAP is subject to assessment, remediation, monitoring, and reporting regulations in the USA’s Comprehensive Environmental Response, Compensation, and Liability Act of 1980 (CERCLA). Details of methods data collected during the assessment, construction, pilot project, full scale treatment, and monitoring phases of the NRAP project are available in reports issued by the EPA [32–34]. These details include site hydrogeology, contaminant levels and distribution, geochemistry, and general water quality. All measurements were done using standard operating protocols as required under CERCLA, including methane emission from the site, measured in the vadose zone and dissolved in site groundwater.

The initial PCE concentration at NRAP was nearly eight million-fold over the maximum contaminant levels (MCL = 0.005 mg/L). The water table is encountered at approximately 1.5 m bgs in the shallow aquifer, which is comprised of two layers, a high-permeability sand/gravel/cobble mixture, which extends to approximately 6.1 m bgs and a 1.2 to 2.0-meter-thick sequence of interbedded fine-grained sands and sandy clay layers from approximately 6.1 to 8.0 m bgs. Beneath this shallow hydrostratigraphic zone is a 6 to 12-meter-thick clay layer that is generally continuous across the site and hydraulically separates the shallow from the deep water-bearing zones. The lithology below of the shallow aquifer consists of sequences of silts and clays interbedded with fine-grained silty sand and sand units.

**Fig 1:**
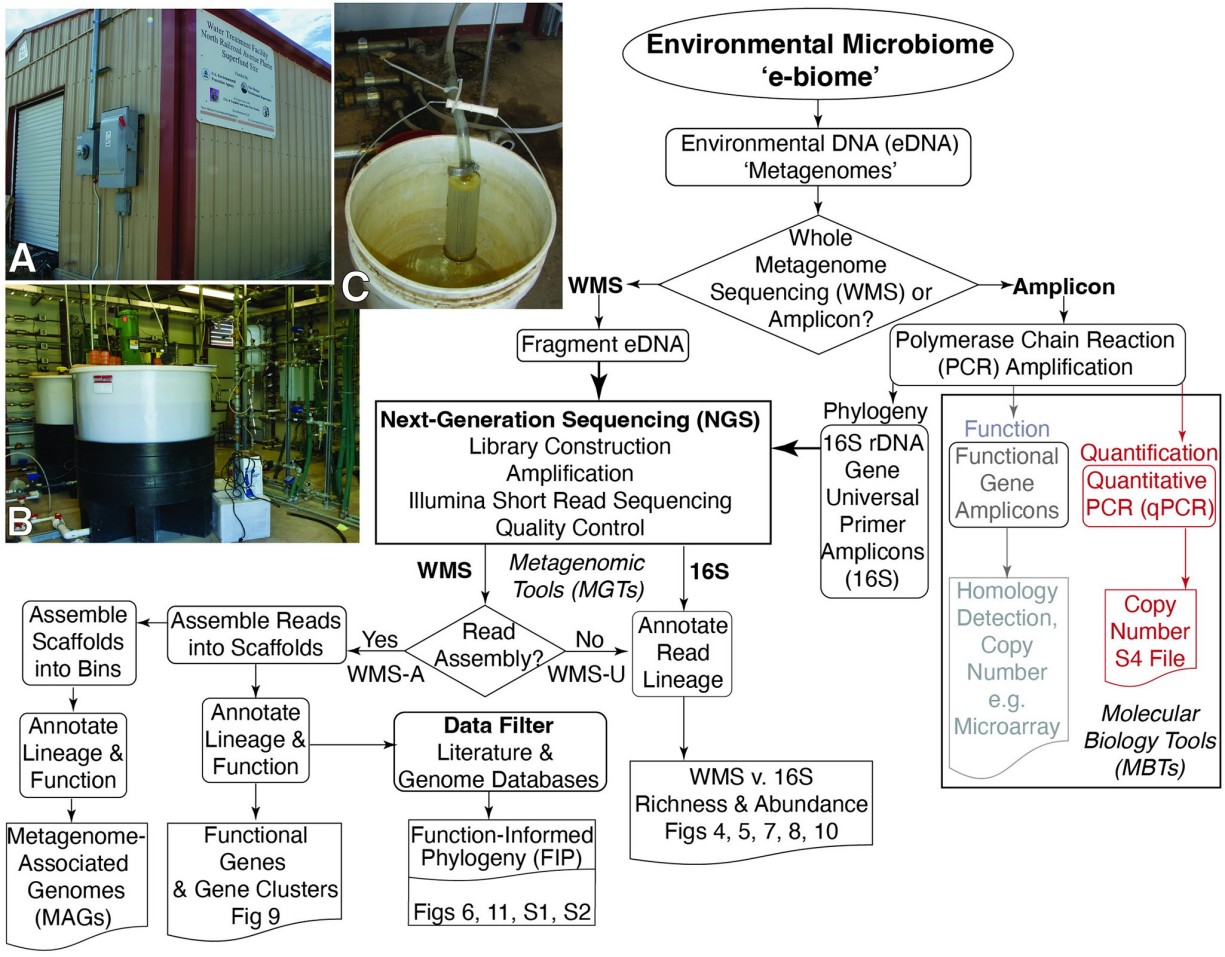
NRAP environmental microbiome metagenomic roadmap. Photo inset: A. The NRAP Water Treatment Facility in Española, NM, USA. B. Interior showing the flow control system and tanks used for amendment mixing and injection. C. On-site e-biome concentration through a filter capsule. Quantitation using PCR (shown in red) a molecular biology tool (MBT) were used in 2006 to detect indigenous microbes capable of ERD. The detection of functional genes (shown in grey) is possible with gene-specific primers. Metagenomic tools (MGTs) involve the use of next generation sequencing (NGS) applied to whole metagenomes or to the pool of amplicons produced with universal primers.

The construction of the remediation system was completed in 2005, resulting in a network of over 100 injection, extraction, and monitoring wells with manifold systems connected to sampling, flow-control systems, amendment mixing, and injection apparatuses (Fig 1A and 1B) [35]. This infrastructure was organized into four treatment areas; the source zone, the hot spot, the downgradient region located in the shallow aquifer, and the deep zone. During the assessment phase in 2006, molecular biological tools (MBTs) were used to determine if TCE and DCE groundwater plumes collocated with the PCE plume were due to microbial action. The results of quantitative polymerase chain reaction (qPCR) revealed the presence of *Dhc*, *Dhb*, and *rdh* genes (S4 File) known to be required for OH respiration. Additionally, phospholipid fatty acid (PFLA) and denatured gradient gel electrophoresis (DGGE) on microbes harvested from groundwater samples in 2006 detected microbial taxa capable of supporting ERD [36].

The combination of hydrological, chemical, and MBT data supported biostimulation by ERD as the most efficacious and cost-effective remedy. A pilot test conducted in 2006 determined the optimal amendments and nutrients and the delivery methods for successful remediation [35]. This was achieved by the injection and recirculation of electron donors and nutrients in-situ to deoxygenate and reduce the aquifer in support of anaerobic biodegradation [37]. Bioaugmentation, where non-native microbes are injected, was deemed unnecessary.

Groundwater was pumped from the aquifer into tanks (Fig 1B), mixed with the selected amendments, reinjected into the aquifer, and then recirculated. All injections included a standard nutrient mix along with the amendments. One month into the pilot test phase, emulsified vegetable oil (EVO) and hydrogen gas (H_2_) were injected into the source area injection wells. Dairy whey was injected into the hot spot wells, which had approximately 10-fold lower concentration of OH [35]. Due to its superior performance as measured by the increase in PCE breakdown products, EVO was selected as the preferred bioamendment and was injected into all pilot-test areas five months after the start of the pilot phase, marking the end of pilot tests and the start of full-scale operations. In a previous paper, we documented the microbial community shift during the pilot tests in the source area, this includes a 12-fold increase in biomass, decreases in aerobic bacteria, and abundance increases in anaerobes post biostimulation [36].

During the full-scale operation additional doses of EVO were injected at 10, 15, and 21 months to maintain sufficient levels of substrate for optimal ERD. At 25 months, ethyl lactate was injected to supply an additional, highly-mobile carbon source. Results from groundwater and soil-gas sampling and analysis, including pH, oxidation-reduction potential, total organic carbon (TOC), and biogas (predominately CH_4_ and CO_2_) production at well headspaces were used to assess the dosing of bioamendments and nutrients throughout treatment. Table 1 provides the NRAP e-biome sampling history in relation to the treatment methodology in the source and hotspot zones.

**Table 1:** NRAP shallow plume treatment and e-biome sampling history.

NRAP e-biome Source	Samples (Dates)	Sample Treatment History	WMS	16S
**Source Area Extraction Well #3 (SAE3)** Lat: 35°59’31”N Long: 106°04’53”W Elevation: 1,703 m Well Depth: 2.89 m	**SAE3-00**: 0 months, Baseline (June 2007)	None	**+**	+
	**SAE3-04**^1^: 4 months (Nov. 2007)	EVO + H_2_ @ 1 month	**+**	**+**
	**SAE3-23:** 23 months (June 2009)	EVO + H_2_ @ 1 month, EVO @ 5, 10, & 15	**+**	**+**
	**SAE3-39**^2^: 39 months (Oct. 2010)	EVO + H2 @ 1 month, EVO @ 5, 10, 15, & Ethyl Lactate @ 25	**+**	**+**
**Hotspot Extraction Well #6 (HSE6)** Lat: 35°59’31”N Long: 106°04’53”W Elevation: 1,703 m Well Depth: 2.93 m	**HSE3-0**^3^: Baseline, 0 months (June 2007)	None	ND	**+**
	**HSE6-04**^1^: 4 months (Nov. 2007)^2^	Dairy Whey @ 1month	**+**	**+**
	**HSE6-23:** 23 months (June 2009)	Dairy Whey @ 1month, EVO @ 5, 10, & 15	**+**	**+**

ND–Not Done

^1^. Listed as SAE3-5 and HSE3-5 in the JGI Integrated Microbial Genomes (IMG/M) database

^2^. Listed as SAE3-47 in the IMG/M database

^3^. Insufficient DNA from the HSE6 baseline was isolated for WMS and only one 16S amplicon run was available.

Prior to remediation TOC was low and PCE was the dominant cVOC. TOC increased as a results of bioamendment dosing and after 16 months all daughter products of PCE degradation were apparent. PCE was mostly not detectable at 38 months and ethene was the predominate VOC [35]. As of 2020, 13 years after bioremediation procedures were implemented, 90% of the PCE at the source area was eliminated and concentrations in the rest of the shallow aquifer, including the hotspot, were below the MCL [34]. The deep zone still contains significant amounts of contamination. NRAP is half-way through the expected 30-year completion. The reports required by CERCLA are a rich source of metadata that provides information on the environmental conditions under which ERD is proceeding. Throughout this report site metadata, including OH and methane concentrations, are merged with the results of metagenomics to develop an improved understanding of ERD in the first 3.25 years of bioremediation.

### Application of metagenomics to e-biomes

The 1986 development of PCR and the 1990 description of 16S ribosomal DNA sequence as phylogenetic markers made possible the 2006 state-of-the-art MBTs capable of predicting the success of biodegradation as a solution for environmental contamination. High-throughput sequencing, a.k.a. next generation sequencing (NGS) became available in 2005 and ushered in a new era in which any DNA sequence can be rapidly and accurately sequenced. In 2007, the National Institutes of health launched the Human Microbiome Project that helped to promote the application of NGS to microbiomes. While MBTs such as qPCR are effective diagnostic tools, metagenomic tools (MGT) reveal the complexity of microbial interactions that support ERD.

WMS involves the random fragmentation of DNA, which is subsequently size selected, primer-adapters are added to both ends, and sequenced then using NGS instruments. Because primer-adapters provide identical priming sites on every fragment from which subsequent amplification and sequencing reactions proceed. Short read (e.g. 150 bp) data can be assembled into regions with matching sequence, producing scaffolds with adequate length for gene identification. If adequate sequence coverage is available, further assembly (binning) yields complete genomes, known as metagenome-associated genomes (MAGs) that can become new reference genomes irrespective of the availability of cultured representatives.

The application of single-cell sequencing also contributes to the documentation of previously unknown diversity, known as the ‘microbial dark matter’ that remains a challenge for taxonomic annotation [38]. Current taxonomic standards require a cultured microbe that represents the taxa to be considered ’validly published.’ Taxa designated as ‘*Candidatus*’ include uncultured reference microbes from MAGs or single-cell reference genomes and have passed quality control measures [39]. Strain designations are initially assigned to microbes in culture, pure or mixed, and can be associated with a function, such as OH respiration. Once the DNA from a strain is sequenced, assembled, annotated, and passes quality control measures, it becomes a reference genome in the databases that are used by algorithms to determine the phylogenetic lineage of new sequence data. While most reference genomes do not have validated functional data in the literature, function can be inferred from the lineage and/or presence of key genes, such as *rdh*.

WMS was used to thoroughly characterize an aquifer in Rifle Colorado, USA that significantly expanded our knowledge of microbes in groundwater systems [40]. This project included multiple replicates of different parts of the aquifer at different time points, yielded 47 new phyla and over 2,500 new genomes using bioinformatic workflows that exploit the volume of data generated by WMS. This including the re-assembly of genomes from short read data [41]. WMS has the advantage of producing phylogenetic lineage, gene product detection, and abundance information from the same dataset. Disadvantages of WMS for bioremediation projects includes the high cost compared to qPCR, the lack of workflows and data filtering protocols that apply to biodegradation projects, and the need for standards to guide environmental engineers accustomed to MBTs.

NGS also provides a significant advance for 16S rRNA gene analysis by allowing PCR amplicons resulting from universal primers to be sequenced *en masse*. PCR has the advantage of requiring a small amount of DNA when compared to WMS. A disadvantage of PCR is that requires *a priori* knowledge of the target sequence so its ability to detect microbes yet to be sequenced is limited. In addition, target molecules can compete for the same primers that can skew abundance estimates. This method is less expensive than WMS and is a suitable replacement for MBTs such as PLFA and DGGE analyses used to characterize the functional potential of microbial communities. While 16S alone cannot confirm the presence of genes important in biodegradation, additional primer combinations specific for genes of interest can reveal their presence. Microarrays that identify *rdh* genes specific to *Dhc* use this strategy and provide a useful diagnostic tool for engineers [42].

Water was filtered at the NRAP water treatment facility (photo insert Fig 1A–1C) and flushed from the filters without applying enrichment protocols. The rest of Fig 1 is the bioinformatics flow used to analyze the NRAP e-biome. For WMS, eDNA was fragmented in contrast to 16S, were eDNA was first subjected to PCR amplification using universal primers. Metagenome fragments and 16S amplicons were subjected to the same NGS techniques, which started with the addition of primer-adapter sequences to both ends of each DNA molecule. Sequencing proceeded from both ends and generated 150 bp reads. After quality read control procedures, the lineage was determined for every read by comparing to databases; amplicons were matched to a 16S rRNA gene database and WMS was compared to a genome database. Two post-sequencing workflows for WMS short-read data were performed, phylogenetically annotated unassembled reads (WMS-U) and reads assembled into scaffolds (WMS-A). With adequate coverage, scaffolds can be further assembled into bins that can become MAG reference sequences. NRAP scaffolds were filtered for function using three strategies. The first involved selecting taxa identified in the peer-reviewed literature. Second, scaffolds were selected that contained coding sequences (CDS) for key functional genes, such as *rdh*, *hdh*, and *mo*. Third, the key gene search was repeated in the IMG/M database [16] to find all instances of genomes containing these genes. The results of these searches were merged with the WMS-U timeline data to identify taxa with the potential to support ERD. We refer to this process as function-informed phylogeny (FIP) to indicate that both literature-validated functional information derived from studies of culturable strains and sequence-derived gene and taxonomic information were considered in the data filtering processes.

FIP facilitates functional analyses on metagenomic data that is too diverse to be assembled into draft genomes. FIP also makes it feasible to predict functions for unculturable microbes. Combining literature-validated functional information with function inferred by the presence of key genes then mapping the abundance changes for each genus, results in the identification of additional taxa involved in ERD.

This report covers the first 3.25 years of ERD in the shallow region of the plume and includes the resequencing of previously reported baseline and four month samples from the source area [36]. New data include source area collected at 23 and 39 months and hotspot samples from four and 23 months that were subjected to WMS and 16S metagenomic analyses. In this report, ’strains’ refer to microbes in culture, either pure or mixed, that can be assigned a function, such as the ability to respire or cometabolize OH. The term ’genome’ indicates fully sequenced and annotated strains as listed in IMG/M [16]. Additionally, the use of OH-respiring genera (OHRG) rather than OH respiring bacteria (OHRB) and COMG instead of cometabolic bacteria (COMB) reflects the focus on genus-level taxonomic identification and allows for the inclusion of *Archaea*.

This observational project explores the potential of WMS to reveal details of a successful ERD project. The first objective is to compare the efficacy of the MBTs used to select ERD as a remedy in 2006 to the MGTs now available. Second, WMS is compared to 16S as methods to improve the understanding of e-biomes capable of ERD. The final objective is to identify candidate microbes and processes inherent in the NRAP e-biome that contribute to the syntrophy required for successful ERD.

## Materials and methods

### Sample collection and DNA preparation

Access to the NRAP site was facilitated by coauthor PAG, NRAP site manager at the time of these sample collections. Site sampling (Table 1) involved the previously described on-site filtration of microbes and direct extraction of eDNA in the lab that avoid enrichment procedures [36]. Briefly, 0.22 μm capsule filters (Cat. #12117, Pall Gelman, Ann Harbor, MI) were attached to the extraction well outlet (Fig 1C), the volume of water filtered was recorded, and filters were transported to the lab on ice with entrained water. Microbes and sediment were backflushed from the filters using 10 mM Tris-SO_4_, the resulting solution was centrifuged (25,000 x g for 30 minutes), and the pellets stored at -20°C. To insure the breakage of cell walls, samples were flash frozen in liquid N_2_ (-196°C) prior to extraction then allowed to thaw at room temperature. DNA extraction and purification was accomplished with the G-nome^®^ DNA isolation kit (Qbiogene Cat # 2010–200, Carlsbad, CA) and the Geneclean^®^ Turbo-Kit (Qbiogene cat #1102–200). DNA quality was assessed with agarose gel electrophoresis and Qubit fluorimetry. Concentration of the eDNA was confirmed via UV spectroscopy. Biomass estimates were based upon the DNA yield per liter of water filtered. The concentrations of all VOCs and methane for each timepoint was extracted from the EPA site report [35].

### Library preparation, sequencing, and bioinformatics workflow

The eDNA was sequenced and analyzed as part of the JGI Community Science Program [43]. The JGI Microbial Genome Annotation Pipeline (MGAP) was utilized for the bioinformatics workflow [41, 44].

Standard Illumina TrueSeq^®^ protocols were used for WMS. The eDNA was fragmented, the 270 bp fraction was selected, and bar-coded primer-adapters were added to each fragment. Only one replicate was available for each sample but includes sequencing from both ends. The samples were pooled and run in a single lane of an Illumina HiSeq-2000 flow cell and paired-end reads of 150 bp were generated using standard operating protocols as recommended by the manufacturer.

The JGI MGAP workflow includes quality control, contamination filtering, assembly, feature prediction, functional annotation, taxon annotation, and binning [44]. The details of taxonomic assignment protocols are provided in S1 File. Briefly, if a strain-level taxonomic assignment cannot be made, then the species level is match is calculated and this process is repeated until a match is achieved. This results in a list of reads identified (IDed) at a variety of taxonomic levels, from strain to domain, or no ID if matching sequences were not found in the database. Reads that remain unidentified at the domain level reflect the abundance of microbial dark matter that was not available in the databases. Data was filtered to include all reads IDed at the genus level for further analysis.

For 16S rRNA gene amplification, eDNA extracts were dispensed into a 96-well plate. Illumina iTAG V4 primers, PCR, and library preparation protocols were followed as recommended by the manufacturer. Sequencing was carried out on the Illumina HiSeq-2000 using the same protocol as for WMS. There were 11 sequencing replicates for the SAE3 samples, five for HSE6-4, four for HSE6-23, but only one was successful for HSE6-0. The 16S bioinformatics workflow details are provided in S1 File.

Raw counts and metadata available through IMG/M and were downloaded from the JGI Genome Portal and imported into the statistical package JMP (v17.0.0, Cary, NC) for further analyses. Each line in the downloaded WMS and 16S raw data files represents a single read, the data were tabulated at the strain level to determine the number of reads per genome. The files for well-timepoint were joined. The scaffold data files with the length, depth, and linage information were joined using the scaffold object identifier (OID) as the key. A list of gene CDS from the combined assembly was downloaded and filtered by searching for keywords (e.g. dehalogenase). The 16S results are reported at the genus-level with a 95% confidence, so WMS-U was tabulated on the genus for comparison. The WMS-U and 16S rRNA datasets were joined using the genus designation as the matching (key) value. The resulting data files subsequent analyses are provided as supplemental material (S2 to S4 Files).

### Taxonomic nomenclature updates

Updating the taxonomic nomenclature was necessary to account for recent changes, including revisions of lineages and the renaming of 42 phyla [45]. Taxonomic lineages are ontologies where any level can have multiple children, but only one parent, making it possible to detect differences by a series of tabulations. The steps in JMP used to update the lineages to ensure that tabulations on any taxonomic level were correct are shown in S3 File. The List of Prokaryotic Names with Standing in Nomenclature (LPSN) was consulted for the most recent assignment [46].

### Normalization to biomass, scaling, and PCoA

The average DNA yield (μg/L) for each timepoint was used as an estimate of biomass that was used to calculate a normalizing factor (NF) for the sample *i* is found as:

$${\text{NF}}_{i}={\text{Yield}}_{\text{i}}/{\text{Yield}}_{\text{i0}}$$ (1)

where Yield_i_ = DNA yield (μg/L) for sample *i* and Yield_i0_ is for the lowest-yielding sample (SAE3-00). Read totals by strain (WMS) and by genus (16S rRNA) were divided by this factor to generate normalized reads that were scaled to reads per million (RPM). This value was used for all subsequent calculations.

To validate the response of the NRAP e-biome to ERD protocols, a principal component analysis (PCoA) on log_10_-transformed RPM data for SAE-3 using JMP. For visualization, genera were filtered to include only those with valid taxonomic assignments, eliminating *Candidatus* and not-yet validly assigned genera. The Genomes On-Line Database (GOLD) [16] was searched to find genomes for which O_2_ requirement is documented; this was used to annotate the PCoA markers by color. For the interactive html version the domain is indicated by marker shape (S5 File).

### Function-informed phylogeny

Taxa with validated ability to degrade chlorinated ethenes in lab studies are listed in the S1 and S2 Tables in S1 File. Metagenomics provides an additional method to identify genomes with this potential by searching for genes in genome databases that code for the central enzymes involved in dechlorination. The term function-informed phylogeny (FIP) is proposed to distinguish using genome database searches from analyses that rely solely on literature-validated comparisons. The filtering and matching processes were done at the reference genome (strain) level. For example, search of all IMG/M data was conducted starting by entering the term dehalogenase into the ’find genes’ function in IMG/Expert Review (ER) [47]. The resulting files were downloaded and joined at the strain-level WMS-U data. Those that increased in any post-remedy timepoint were considered to have functional potential, regardless of current literature validation. Genome lists were tabulated on the genus level for visualization, thereby including only those strains that have the selected function. FIP avoids the limitation of relying solely on genus-level identification of OH-respiring taxa, since some species and strains within a genus harbor *rdh* genes, while others do not. The 16S data does not make this distinction but the results are shown in the FIP figures for comparison. FIP provides a means to mine e-biome microbial dark matter for novel bioremediation potential. Results of searches are provided as supplemental information (S4 File).

To test the hypothesis that dark oxygen production may support aerobic cometabolism, *cld* and *nod* gene annotations in the NRAP scaffolds were combined with literature-validated genera identified in other subsurface microbial studies [31]. The resulting data set was filtered to include only microbes that increase in abundance after baseline in at least one timepoint.

### Data visualization

The standard visualization method used for 16S rRNA gene amplicons is the stacked bar chart with each color representing a different taxon and the abundance represented by the height of the segment. This approach does not work well for WMS results since the number of different taxa detected are significantly greater compared to 16S results, making it unreasonable to distinguish taxa by color. Additionally, comparison between samples taken at different times can be difficult to discern on stacked bar charts when taxa exhibit large changes in abundance. Cells plots solve these problems by using a color gradient to indicate relative changes in abundance. Each row is a different taxon with the name next to the row and timeline data are displayed as columns. Cells plots are sorted on the basis of abundance at baseline, from high to low. Stacked bar graphs were used for cVOC data and shaded line graph for methane data. The data files used to create all plots and graphs provided in Excel format (S3 File). Image files were exported into Adobe Illustrator (V24.0.1) for markup and file type conversion.

## Results

### Overview

A benefit of sampling at a site with remediation infrastructure is that the flow rates needed for injection, recirculation, and extraction also provides enough flow and pressure to collect e-biomes using filter capsules designed for sterilization of commercial products. Capsules enclose a fluted filter with a surface area that can support the flow and pressures supplied by the remediation equipment. The drawback to these capsules that they are not designed for the recovery of microbes from the filter. However, back-flushing and centrifugation protocols proved effective to recover microbes captured on the filter. At 0 months (baseline), more than 400 L of groundwater were filtered without clogging during sample collection. In later timepoints, some filters clogged after as little as 6.25 liters of flow (Fig 1A). Biomass, as measured by μg DNA per liter exhibited an inverse relationship to the volume of water filtered, indicating that increased biomass decreased the time to reach filter capacity. *Dhc*, *Dhb*, *Desulfuromonas* and *Desulfitobacterium* were detected in the baseline sample with WMS-U, consistent with the original qPCR metadata collected during the assessment phase (S4 File). The number of reads IDed ranged from 0.2% in the baseline to 2.3% to 5.6% at all other time points (Fig 1B). Average scaffold size was greatest at baseline then decreases about 100-fold (Fig 1C). The number of scaffolds was 100-fold lower in the baseline, compensating for the increased size. There are no baseline scaffolds for *Dhc* or *Dhb* despite their detection by qPCR in the assessment phase, but both are represented in scaffolds at four, 23, and 39 months. A total of 2.6 x 10^6^ scaffolds resulted from the combined assembly, 0.1% are assigned to a genome, 54% were assigned to a genus, and 35% had no taxonomic ID. Over 4 x 10^6^ gene CDS were contained within all NRAP scaffolds, representing 30,940 genes, including 161 Archaeal and 1,711 16S rRNA small subunit genes. Only 9.7% of the small subunit genes were assigned to a genus with WMS-A, congruent with the observation that only 3% of all WMS-U reads were assigned to a lineage.

Taxa richness, as determined by the number of taxa present at each phylogenetic level, was greater in WMS data than in the 16S data (S7 Table in S1 File). WMS analysis detected 3 times as many taxa at the phylum to the family level, this increases to 6 times at the genus level. While taxonomic richness was generally highest at baseline, this is most pronounced in WMS data, with 51 phyla represented prior to remedy application decreases 12% (44) in later months. The There is a 16% decrease in the number of genera after remedy application.

**Fig 2:**
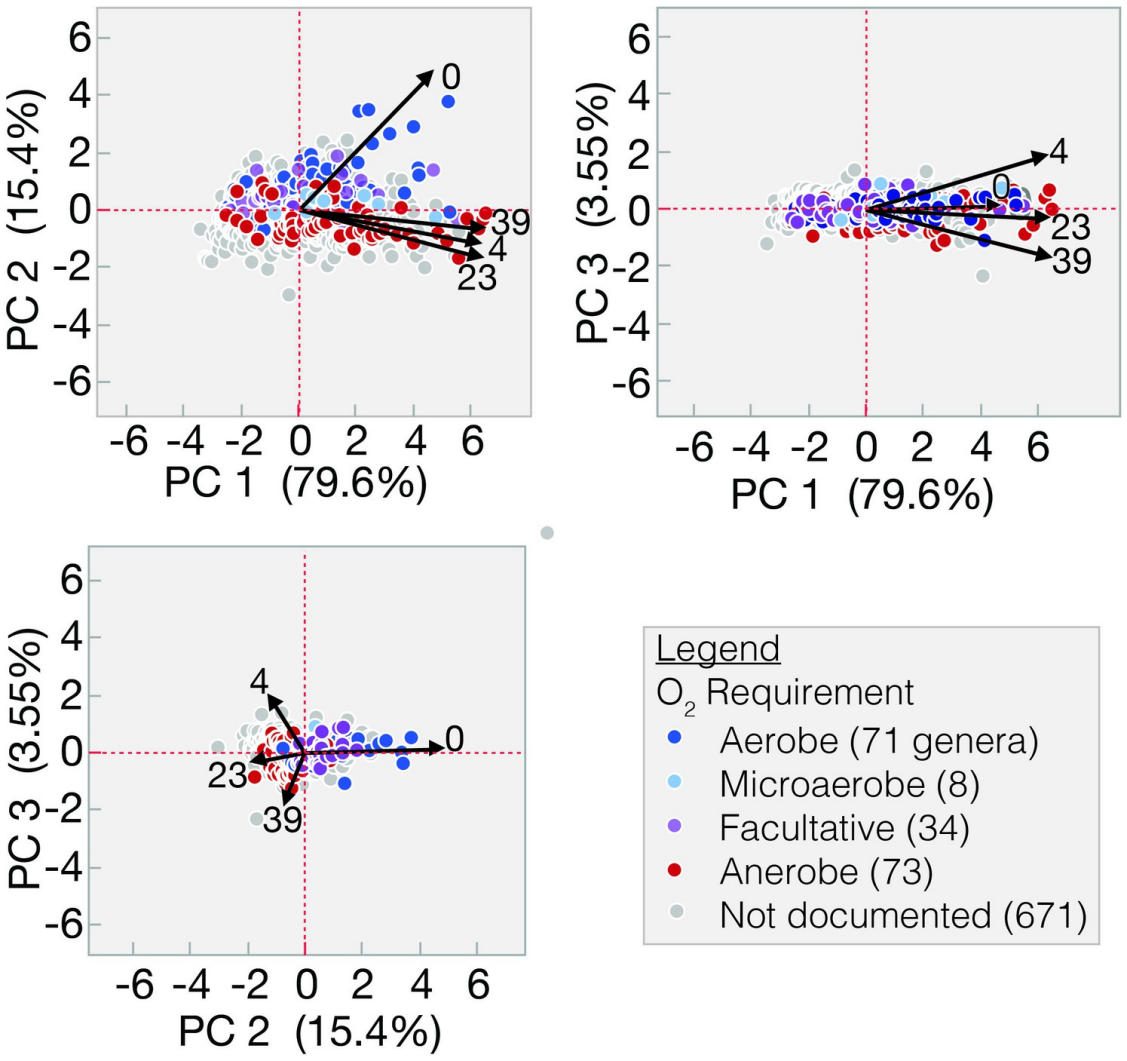
PCoA plots for SAE3. Of 857 validly assigned genera (no *Candidatus*), 186 have documented O_2_ requirements. S5 File is an interactive htlm plot for PC 1 and 2 that is limited to genera with known O_2_ requirements. Domain-level annotations are indicated by marker shape Pointing at any marker in S5 File brings up a window that identifies the genus.

PCoA analysis (Fig 2) of the SAE3 timeline demonstrates the shift in the microbial community based on the RPM and also in relationship to O_2_ requirement.

### Metagenomics of ERD: From domain to strain

Domain, phylum, class, genus, and strain level analyses reveal additional details of the microbiome shifts during ERD. The number of genera attributed to *Archaea* is 113 in WMS-U and nine in 16S. Fig 2 are domain-level comparison of WMS-U and 16S compared with methane concentrations.

**Fig 3:**
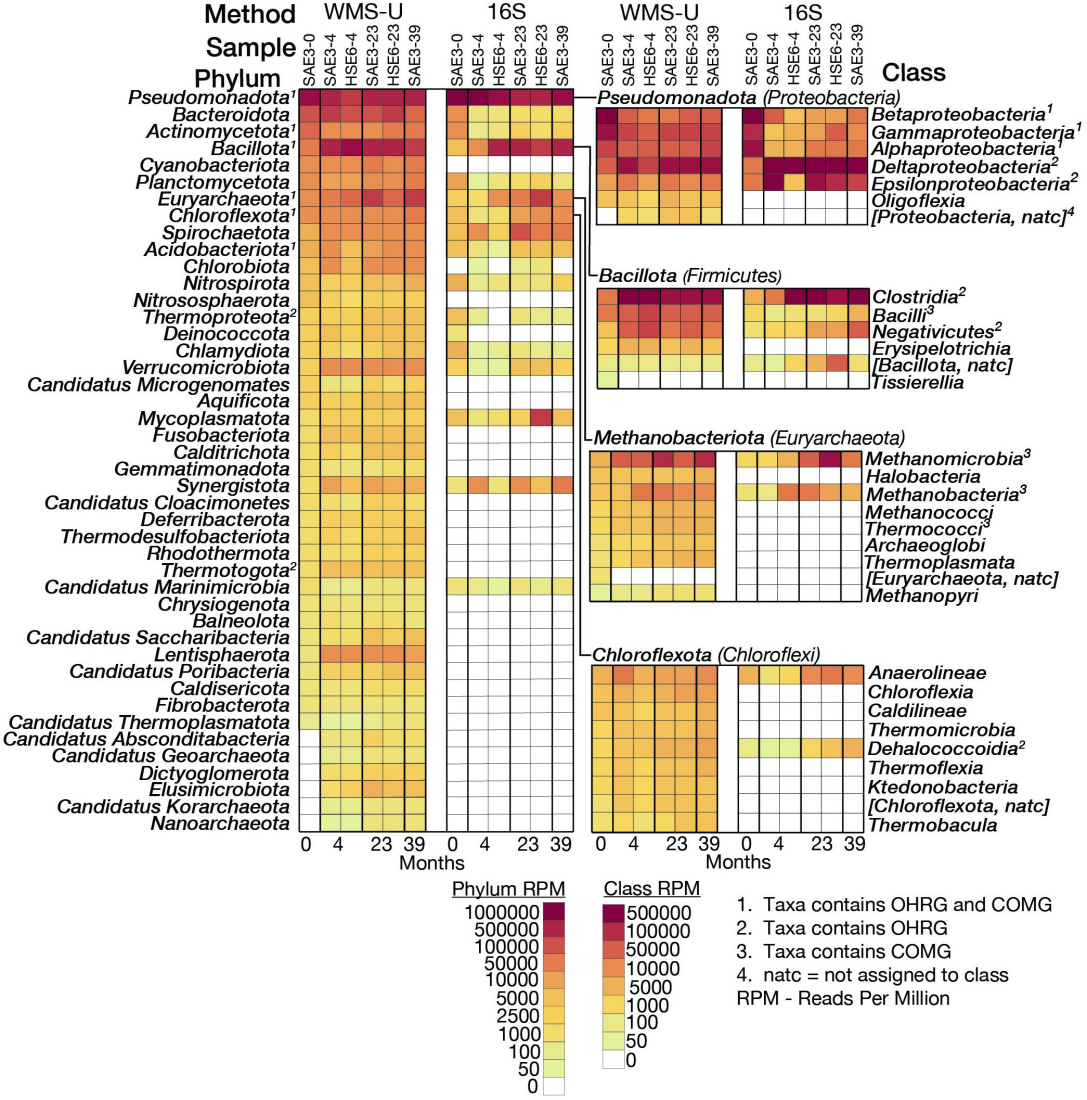
Phylum and selected class-level abundances. Cell plots are sorted from high to low abundance at baseline. WMS-U and 16S rRNA data are shown for six samples, including two samples for four- and 23-month sampling events. OHRG and COMG classification include only literature validated taxa (S2 and S3 Tables in S1 File).

Overall, 57 phyla were detected by WMS-U, 19 were detected by 16S (S7 Table in S1 File). *Candidatus* phyla represented 35% of the phyla detected by WMS-U and 0.6% of the reads. Thirteen of the 57 phyla were found only in the baseline, representing 1.3% of pre-treatment reads, 12 of these were *Candidatus* (S4 Table in S1 File). Phyla detectable after baseline are shown in Fig 3, along with selected class-level abundances and notation indicating if the taxa is known to contain OHRG and/or COMG. For example, the phylum *Chloroflexota* (lower right hand cell plot) includes the class *Dehalococcoidia* that is detected by WMS and 16S. This class is notable because it contains two important OHRG, *Dehalococcoides* and *Dehalogenimonas*.

**Fig 4:**
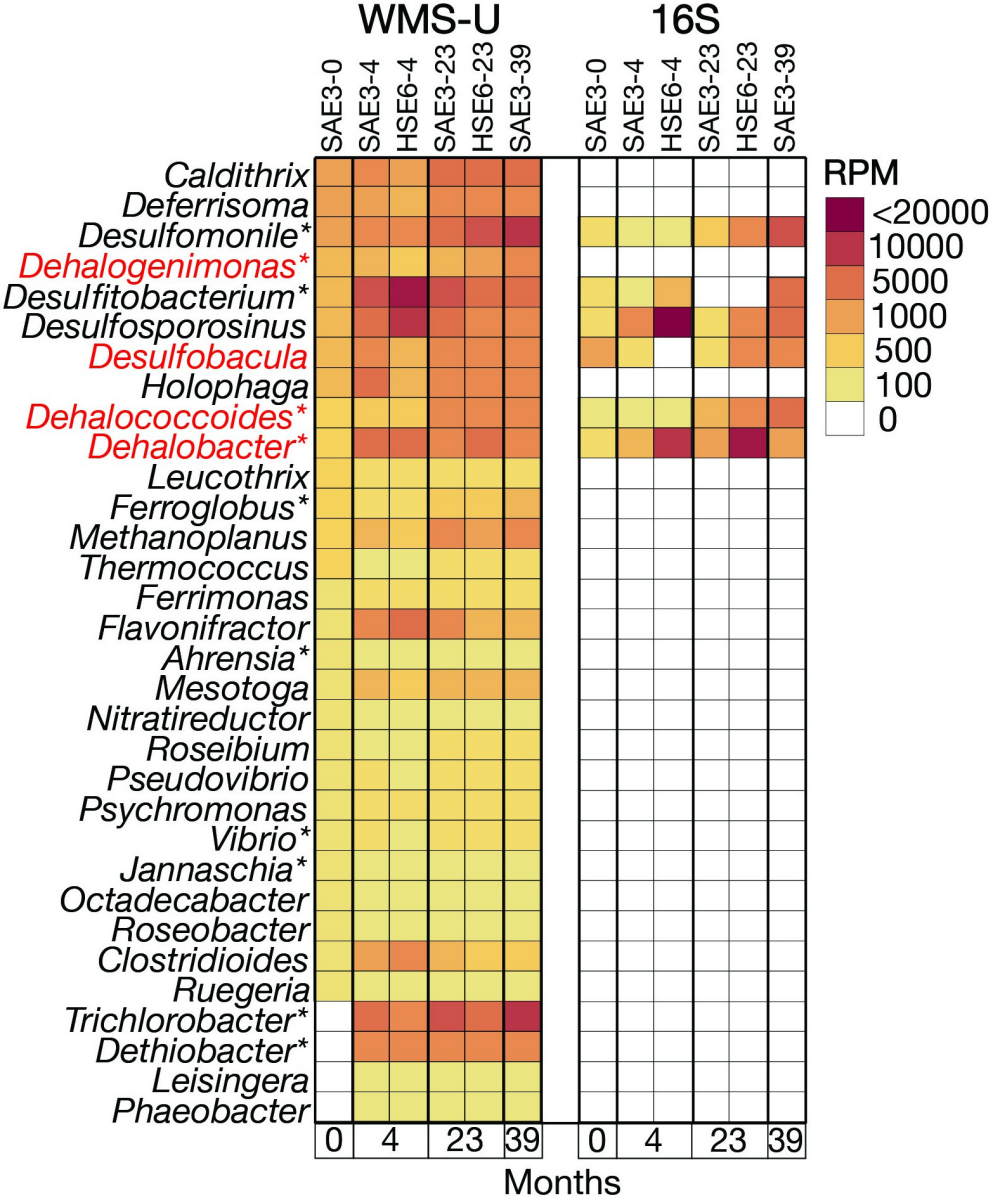
FIP-selected OHRG at NRAP. These genera increase in abundance at NRAP and have *rdh*-containing genomes in IMG/M. The red font indicates that *rdh* -containing scaffolds were detected at NRAP and asterisks indicate literature-validated OHRG (S1 Table in S1 File).

The results of searching IMG/M for *rdh* genes reveals 2,742 genomes that encompass 219 genera but only 30 are validated in the literature as OHRGs. Ninety-eight *rdh*-containing genomes are represented at NRAP, 57 increased in abundance overtime and represent 32 genera (Fig 4).

**Fig 5:**
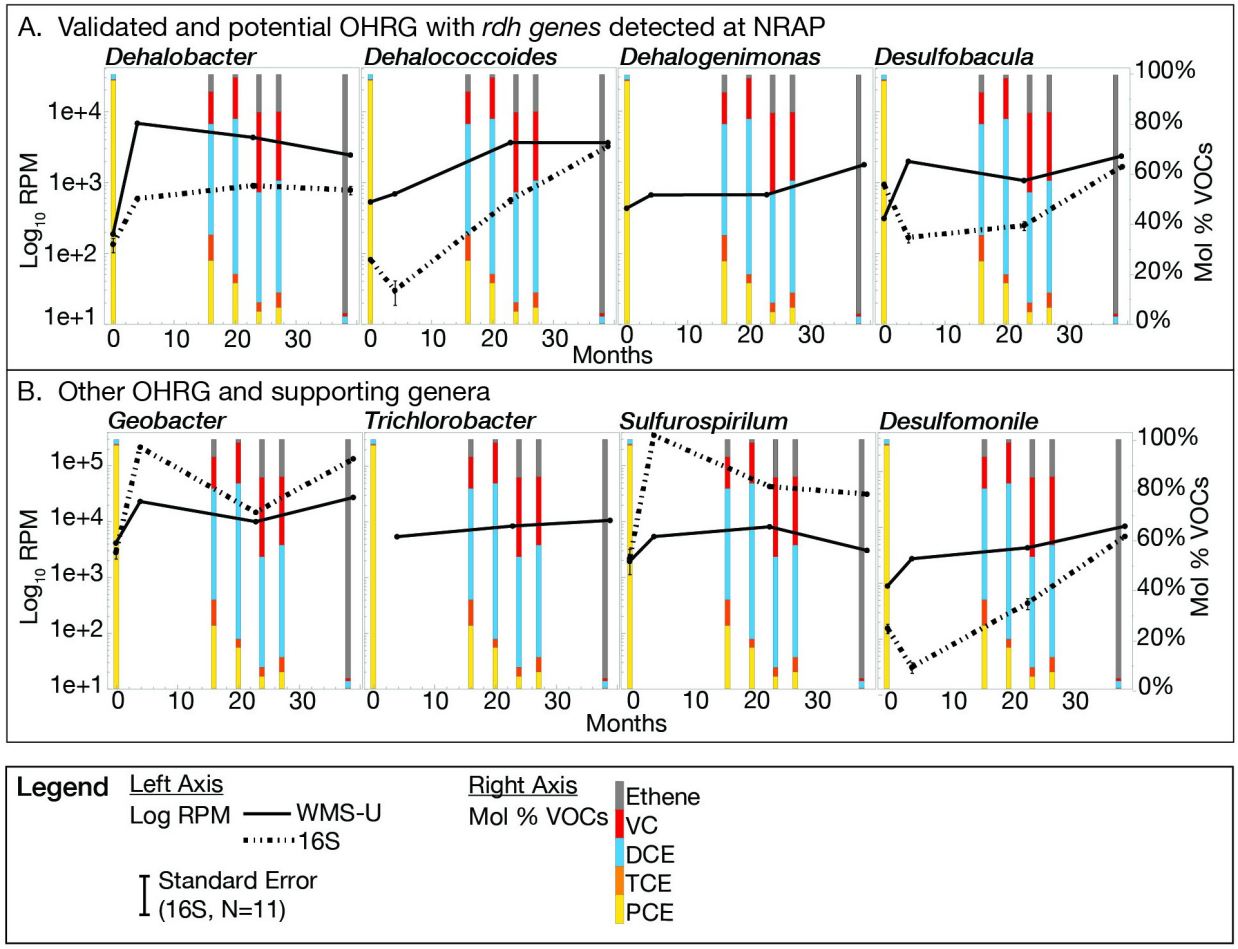
Selected OHRG WMS v 16S. The standard errors for the 16S rRNA gene amplicons are shown, but most are too small to be discernable. Note that the RPM range is greater in B than the other graphs.

A comparison of genus-level WMS and 16S results for selected OHRGs and supporting genera are shown with respect to OH concentrations (Fig 5). Each WMS sample is represented by one paired-end sequencing run while the 16S is based on 11 replicates. WMS did not detect *Trichlorobacter* at baseline and was not distinguished by 16S rRNA. *Dehalogenimonas* was not detected at any timepoint by 16S rRNA.

**Fig 6:**
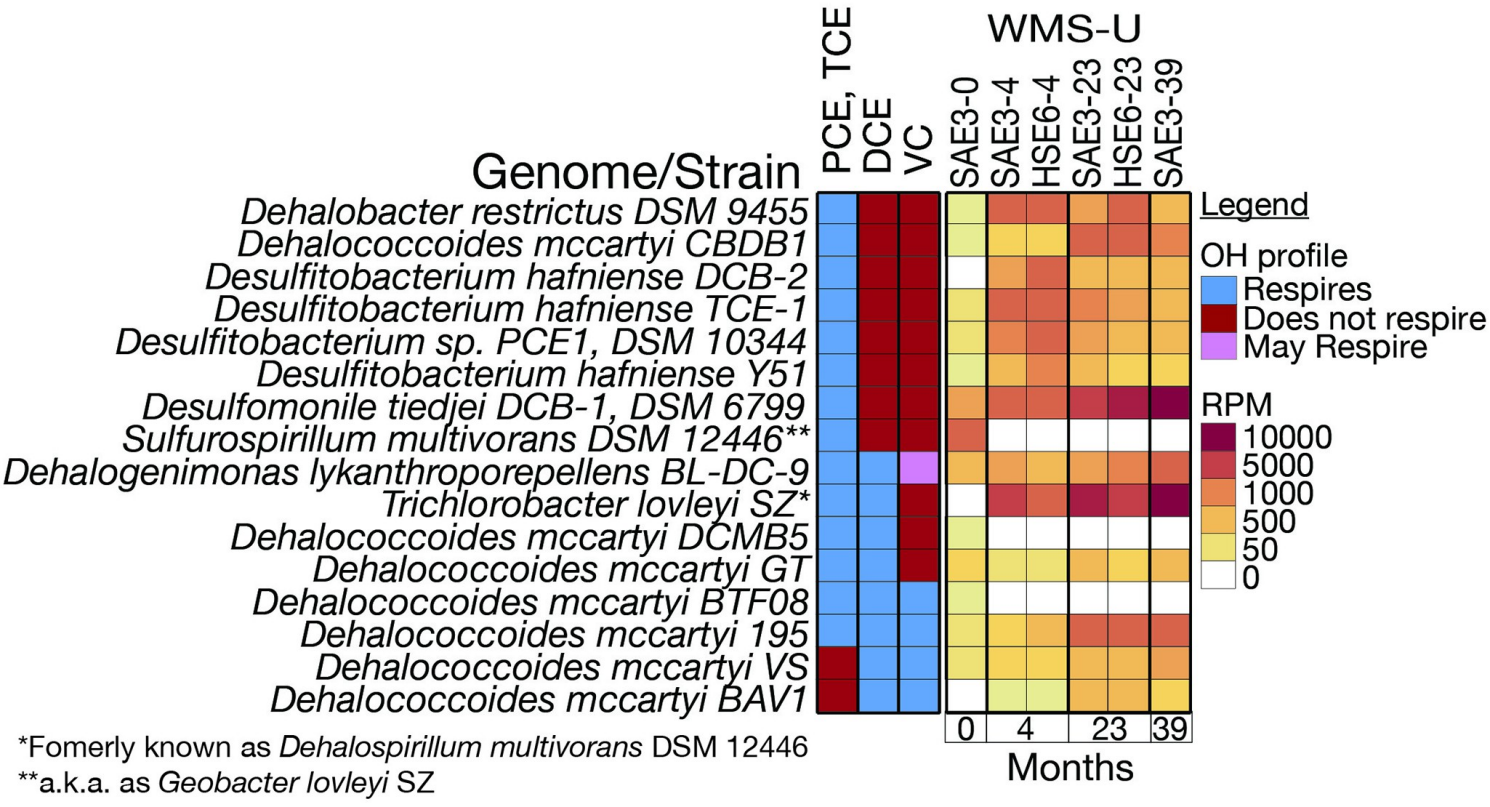
Strain-level OH respiration and abundance profiles. Literature-validated OH-respiring strains combined with abundance profiles demonstrates the pathway for PCE degradation at NRAP. References for the OH-profiles are provided in S6 Table in S1 File.

The profile of cVOC respiration potential (Fig 6) is based on the functional validation of strains in the literature [21, 48]. Combining the respiration profile for each strain with its abundance at NRAP demonstrate that PCE and TCE can be respired by 14 strains, DCE by seven, and VC by three, *Dhc*. *mccartyi 195*, *BAV1*, and *VS*. A new strain, *Candidatus Dehalogenimonas etheniformans GP*, degrades VC [48], but it was not entered into the database until after this data was run. The *Sulfurospirillum* strains at NRAP lack *rdh* genes and are not known to respire OH.

### WMS-A: NRAP scaffolds and bins

**Fig 7:**
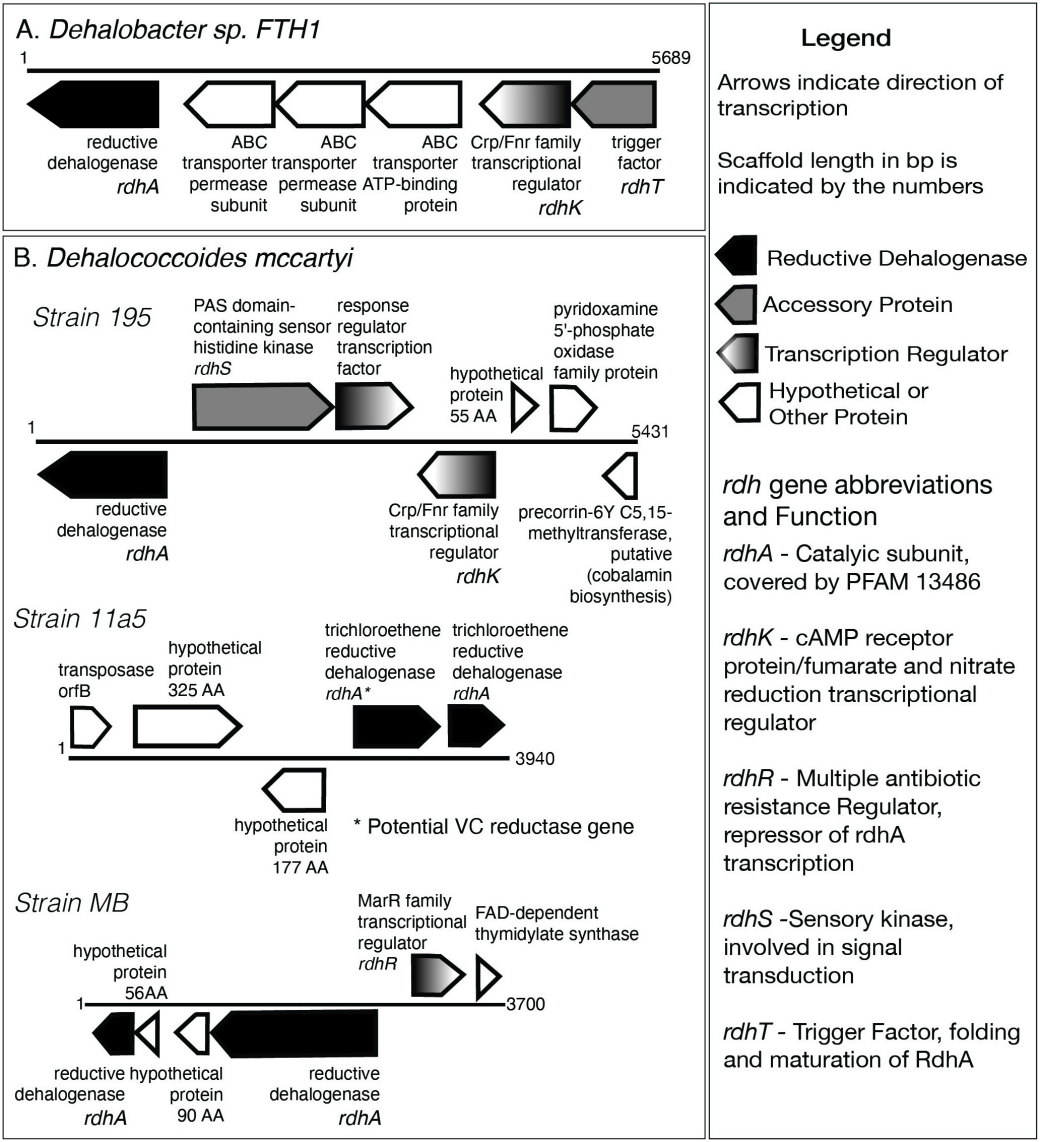
Representative scaffolds containing *rdh* gene clusters. Scaffolds are the result of a combined assembly that includes reads from all timepoints and both wells. Details for all *rdh*-containing scaffolds are in S8 Table in S1 File.

A major benefit of WMS is that the key gene products for ERD can be detected, which is not possible with 16S. The combined assembly detected 390 scaffolds with at least one dehalogenase gene, including 20 *rdh* genes, representing five genera (S7 Table in S1 File). *Dhb*, *Dhc*, *Dehalogenimonas* and *Desulfobacula* include *rdh*-containing scaffolds at NRAP. *Desulfocarbo* also has a *rdh*-gene containing scaffold but was not detected by WMS-U or 16S. Scaffolds shown in Fig 7 include *rdh* and genes for accessory proteins necessary for OH-respiration.

Further alignment of scaffolds by binning produced 127 bins. Of these, four were of high quality, 14 were medium quality, and 109 were of low quality. Taxonomic lineages for the high-quality bins include the Archaeal family Methanoregulaceae, species *Methanobacterium formicicum*, bacterial phylum *Candidatus Omnitrophota*, and the chemolithoautotroph *Sulfuricurvum kujiense*. Additional details are provided in S10 Table in S1 File. Coverage is too low to establish any MAG reference genomes.

### Anaerobic and aerobic cometabolism, dark O_2_ potential

**Fig 8:**
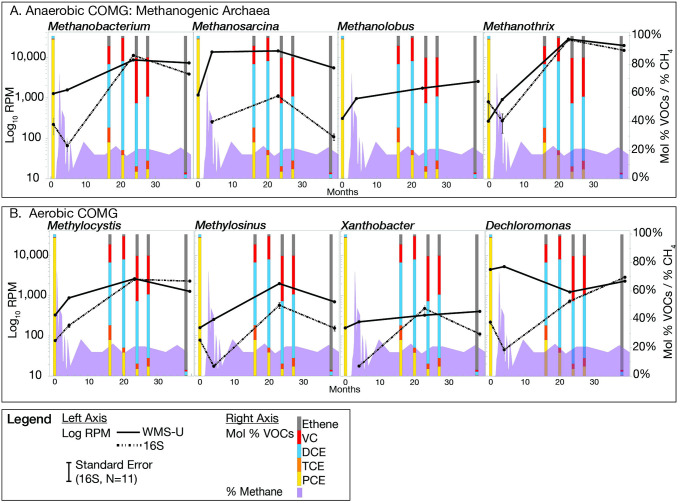
Selected COMG. Methane levels are included since both methanogens and methanotrophs can modulate methane level. A. Methanogenic *Archaea*. *Methanosarcina* is not detected by 16S at baseline and *Methanolobus* is not detected by 16S at any timpoint. B. Genomes with *mo* genes in the IMG/M database and have the potential to oxidize TCE and includes methanotrophs *Methylocystis* and *Methylosinus*. *Xanthobacter* is not detected at baseline by 16S.

The increase in *Archaea* indicates that anaerobic cometabolism occurs as a result of ERD. The abundances of selected methanogenic *Archaea* are shown in Fig 8A.

To determine whether WMS provides evidence for the involvement of aerobic cometabolic pathways, IMG/M was searched for microbes with *mo* gene annotations and the phylogenetic data joined with NRAP abundance data. There are 650 instances of *tmo* and *mmo* genes listed in IMG/M in 539 genomes, 78 of these are found at NRAP and represent 40 genera. Not all of these microbes are currently classified as methanotrophs but harbor the potential to oxidize TCE. The 19 shown in S1 Fig in S1 File increase in abundance over baseline in at least one sample. Fig 8B are examples of genera capable of oxidating TCE due to the detection of *mo* genes at NRAP.

**Fig 9:**
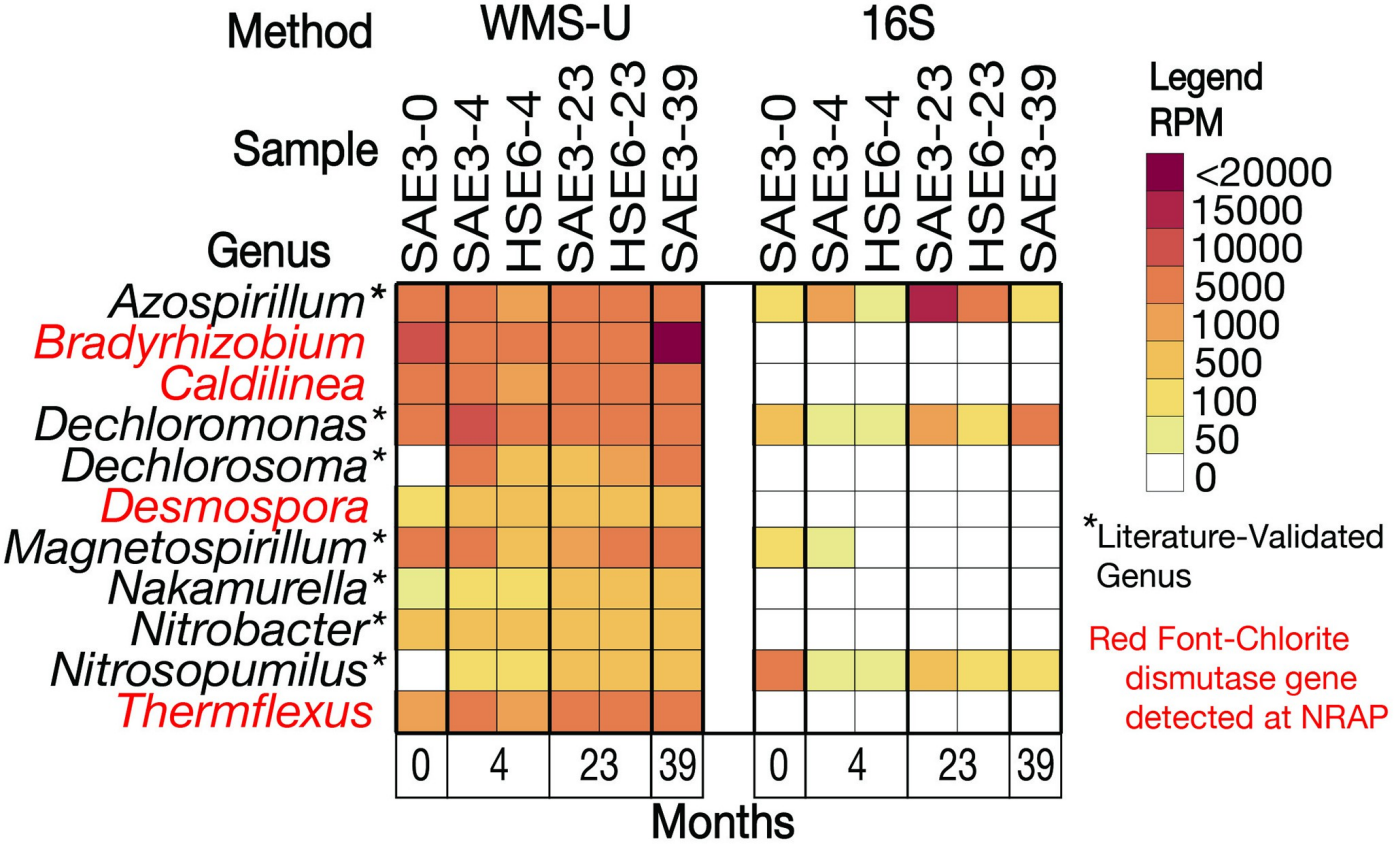
Dark O_2_ producers at NRAP. These genera exhibit an increase in abundance in at least one sample after baseline. Red font indicates that the *cld* gene is detected in an NRAP scaffold. Bold font indicates that dark-O_2_ producing gene products (*cld* and/or *nod*) detected in other groundwater studies [31].

The source of O_2_ to support anerobic cometabolism may be at the soil/water interface but an alternative explanation is that O_2_ is generated by microbes containing dismutase genes. Fig 9 provides evidence for the availability of dark O_2_ at NRAP, these genomes either contain chlorite and/or nitric oxide dismutase genes at NRAP or are found in other anerobic aquifers [31].

Oxidation products and subsequent byproducts can become substrates for *hdh* gene products and there are 212 *hdh* genes detected at NRAP that include haloalkane, haloacid, and haloacetate dehalogenases (S4 File). These genes were distributed among 48 genera that increase at least one timepoint at NRAP. *Desulfobulbus*, *Geobacter*, *Desulfosporosinus* had the highest number of haloalkane dehydrogenases, at 21, 12 and 11, respectively. Other genera with *hdh* annotations at NRAP include *Trichlorobacter*, *Sulfurospirillum* and *Desulfomonile*, *Methanothrix*, *Methylocystis*, *Dechloromonas*, and *Xanthobacter*. The remainder of genera with *hdh*-containing scaffold at NRAP are shown in S2 Fig in S1 File.

## Discussion

### Overview

Sampling a Superfund site has issues not encountered in laboratories, such as hydrogeologic heterogeneity, irregular and unsymmetrical contaminant distribution, temporal and spatial variability in the groundwater levels gradients, site access and dependence on extraction well function at sampling time. For example, HSE6 was not operational for the 39-month sampling event. Additionally, EPA regulations prevent the removal from the site the large volumes of water required to account for the low biomass environments, especially those that exist prior to remedy application. The optimization of on-site filtration methods for WMS methods is essential for field studies where transporting large volumes of water and sediment is a regulatory violation or is impractical.

**Fig 10:**
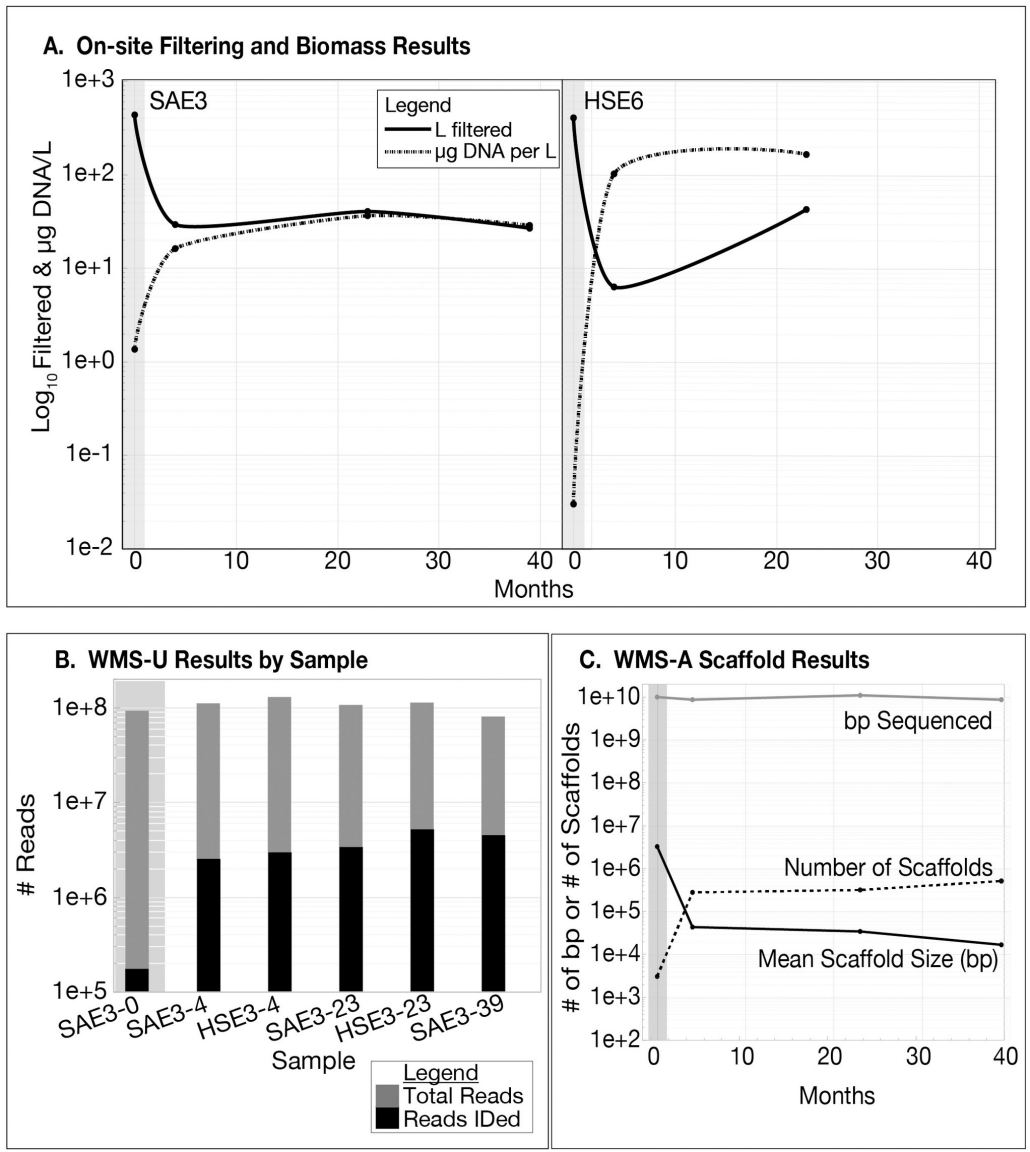
NRAP sampling and sequencing overview. The baseline (0 months) is shaded and all Y-axes are log_10_ values. A. Liters of water filtered on-site and biomass as measured by DNA yield from each sample. B. Sequencing depth and WMS-U identification. C. Sequencing depth and scaffold profiles for well SAE3 over time.

The volume filtered on-site for each sample is negatively correlated to the biomass as measured by μg DNA/L (Fig 10A), demonstrating that clogging was due to an increase in biomass rather than an increase in just sediment. HSE6 had the highest biomass after four and 23 months, which is likely related to the initial pilot-test treatment with dairy whey during the pilot phase and subsequent switch to EVO. SAE3 was exposed only to EVO.

From the environmental engineering perspective, the baseline is critical since it is used to determine the remedy protocol, but the low biomass encountered prior to biostimulation makes it the most challenging sample to obtain. For example, HSE6-0 did not yield enough DNA for WMS and only one 16S replicate was obtained (Table 1). A method to estimate the biomass of the water sample that can be done on-site and then used to determine the volume of water that contains adequate DNA for WMS is in development (unpublished data).

Although the same amount of sequence was obtained for all timepoints (10^10^ bp), there are approximately 10-fold fewer WMS-U reads IDed at baseline (Fig 1B). The high number of reads not identified overall (97%) indicates that there is a large portion of microbial dark matter in the NRAP e-biome for which reference genomes were not yet available [36]. This reflects the state of the databases at the time of analysis rather than a problem with the analysis. As databases become more complete, the percentage of reads that can be IDed will increase. This requires strict data management procedures to ensure that legacy DNA sequence data can be compared to databases into the future.

The number of scaffolds was 100-fold lower at baseline, but this is compensated for by the increased average scaffold size (Fig 1C). The absence of *Dhc* and *Dhb* at baseline in WMS-A indicates that the assembly algorithm needs to be refined for a low biomass/high richness sample. Due to this discrepancy, only the unassembled reads (WMS-U) data was considered for timepoint analyses. Prior to biostimulation there was lower biomass (Fig 1A), more uncharacterized diversity (Fig 1B), and the fraction that was IDed is the richest based on the number of taxa (S7 Table in S1 File). This is the expected response of e-biomes to ERD protocols; taxa that can adapt to anaerobic reduced conditions have a selective advantage over other members of the diverse consortia that existed at baseline. PCoA analysis (Fig 2) reflects these changes, confirming the previously observed response to ERD [36]. This is also consistent with the oxidation-reduction potential measured during site monitoring [35].

### OH respiration: WMS v. 16S

**Fig 11:**
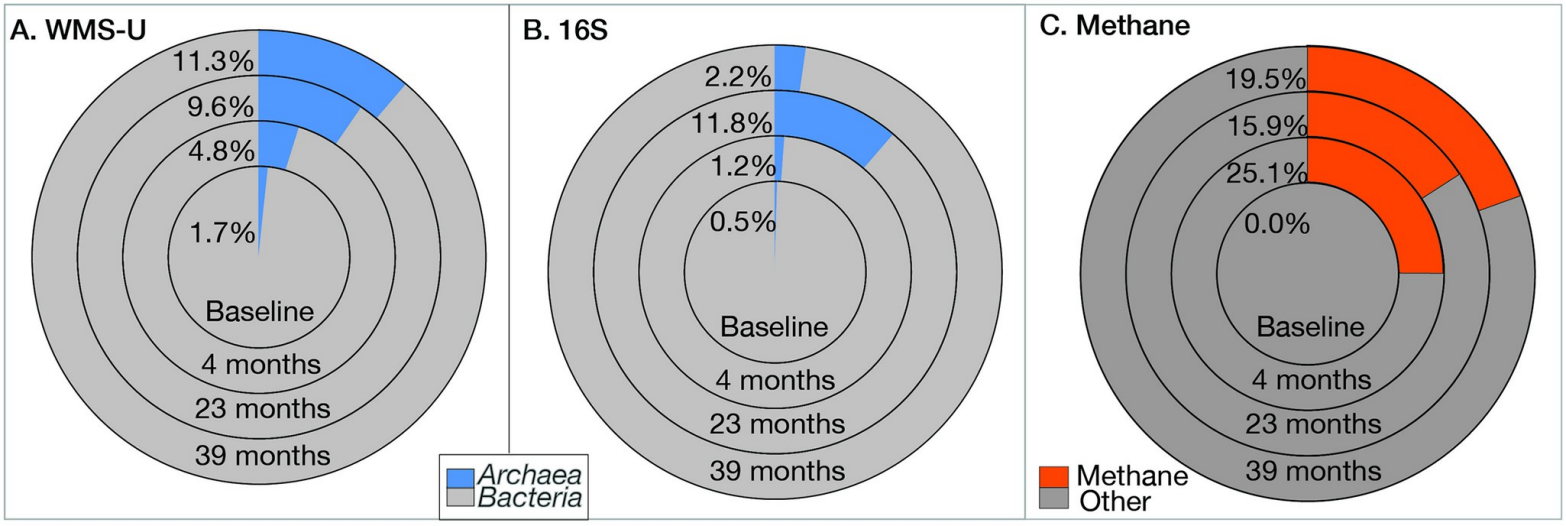
Domain-level abundance and methane saturation. WMS-U and 16S amplicons detected *Archaea* at baseline but methane was not detected until after the start of remediation.

Both 16S and WMS document the increase in *Archaea* after remedy application that was consistent with the increase in methane measured at the well heads (Fig 11). WMS coverage of the phylum *Methanobacteriota* (a.k.a. *Euryarchaeota*) was much greater than 16S (Fig 3) both methods revealed increases in classes *Methanomicrobia* and *Methanobacteria*. The presence at baseline of methanogenic *Archaea* indicates the potential for anaerobic cometabolism but also predicts that contingency plans to mitigation onsite methane build-up are necessary. The abundance increase is most pronounced for *Methanosarcina* (Fig 8A), which is also capable of reverse methanogenesis under some conditions, in which CH_4_ is converted to CO_2_ [49]. *Methanothrix* is not yet a validated COMG but its abundance profile is consistent with the other methanogens (Fig 8A), supporting its role in aerobic cometabolism. The ability of these microbes to degrade OH via transition-metal cofactors [50] and to produce methane is an important consideration when balancing successful remediation with the potential of methane release.

Phylum-level abundance profiles fit expectations for a reduced dechlorinating environment (Fig 3). Although *Pseudomonadota* decreases in abundance over time, both WMS and 16S document increases in *Deltaproteobacteria*, which includes *Geobacter*, and *Epsilonproteobacteria*, which includes *Sulfurospirillum*. *Bacillota* class *Clostridia* (including *Dehalobacter*) exhibits increased abundance. *Chloroflexota* abundance increases, which was expected since *Dehalococcoidia* is the only class containing OHRGs capable of complete respiration of PCE. The second highest abundance at baseline is *Bacteroidota*, which increases at NRAP. The presence of *Bacteroidota* is associated with improved dechlorination of TCE contaminated site undergoing bioaugmentation with KB1 [51]. *Bacteroidota* also contains halogenating enzymes, as do *Verrucomicrobiota* and *Lentisphaerota* [8], linking ERD is linked to the biogeochemical Cl cycle.

Merging literature-validated OHRG with *rdh*-containing genomes from IMG/M indicates that only 8.2% of *rdh*-containing genomes are validated chlorinated ethene reducers, reflecting the diversity of substate specificities that exist for Pfam13486 but also highlighting the need to expand OH respiration testing. Cryptic functions may also exist with amino acid profile of PFAM 13486, increasing the likelihood of misclassification. The 34.4% of literature-validated OHRG that do not yet have *rdh* in any reference genome may be attributable to cryptic taxa in cultures used for testing and/or cryptic pathways for OH degradation. Taxa may harbor *rdh*-containing extrachromosomal elements that escape genome assembly. Validated OH respiring genera at NRAP include *Dhc*, *Dhb*, *Dehalogenimonas*, *Desulfomonile* and *Trichlorobacter*. WMS-A provides evidence for two new candidates, *Desulfobacula* and *Desulfocarbo*, due to the presence of *rdh*-containing scaffolds at NRAP (S8 Table in S1 File). The results for *Desulfobacula* indicate an increase from baseline to 39 months (Fig 5A) but the ability of this *Deltaproteobacteria* to respire OH remains to be validated.

*Dhb* increases rapidly in the first five months, while *Dhc* exhibited a slower initial increase in WMS and decreases in 16S. Both methods indicate *Dhc* increases as VC increases (Fig 5A), consistent with results from microcosms originating from OH contaminated groundwater in which *Dhb* dominated during the presence of PCE, TCE, and DCE (also ethanes). *Dhc* increased as VC became available [13, 52]. Although *Dehalogenimonas* is not detected by 16S the WMS results do not rule out a role in OH respiration at NRAP. The *Dehalogenimonas* strain capable of VC degradation was not in the database at the time of analysis so its role in complete dechlorination at NRAP is unresolved. This strain was detected in samples from the deep zone after 16 years (unpublished data).

*Geobacter* is one of the most abundant genera at NRAP, despite the recommended taxonomic change moves *rdh*-containing genomes into the genus *Trichlorobacter*. *Geobacter* is known for interactions with other microbes, including the transfer of electrons along and the production of cobalamin that can be transferred to cobalamin auxotrophs, such as *Dhc* [18, 53]. *Sulfurospirillum*, *Acetobacterium* and *Desulfitobacterium* are also cobalamin producers that increase at NRAP [19, 20]. *Desulfomonile* is a validated OHRG [54]. All eight genera in Fig 5 also harbor *hdh* genes, indicating that bacteria can have multiple roles in the transformation of chlorinated solvents. *Geobacter* and *Sulfurospirillum* exhibit a higher abundance in 16S than WMS-U, which is reverse of most other comparisons and provides evidence for amplification bias. At four months, 16S analysis results revealed large increases in the abundance of some taxa including *Geobacter* and *Sulfurospirillum*, relative to that of other taxa. WMS analysis results were proportionate. However, 16S results for other taxa including *Methanothrix*, *Methanobacterium*, and *Desulfomonile* (Fig 8A), *Dechloromonas*, *Methylosinus* (Fig 8B), and *Desulfobacula*, and *Dehalococcoides* (Fig 5A) exhibited a decrease in abundance contrary to WMS results from the four-month eDNA samples. These results suggest that high abundance taxa, such as *Geobacter* and *Sulfurospirillum*, can outcompete other taxa for universal primers.

Combining known OH-degradation profiles of strains with their abundances at NRAP reveals a pathway for the complete respiration of PCE to VC (Fig 6). The presence of *Dhc mccartyi* 195 reads at baseline in WMS-U confirms the original qPCR results that predicted the success of ERD at NRAP.

### Gene clusters and MAGs

The *Dhb* and *Dhc* scaffolds shown in Fig 7 contain gene clusters with at least one *rdhA* gene and accessory genes whose products are necessary for RDH [55]. *Dhc* strain 11a5 contains a potential VC reductase gene consistent with the qPCR results and a transposase open reading frame (OrfB). This region has the potential to be relocated to other regions in the genome, including extrachromosomal elements that can eventually be inserted into to other genomes [56]. The *MarR* transcriptional regulator is transcribed in the opposite direction from the *rdhA* genes in *Dhc* strain MB, consistent with its proposed role as a repressor of *rdh* gene transcription in *Dhc* [57]. These scaffolds are not complete operons but corroborate findings that multiple *rdh* genes exist in the dechlorinating microbial consortium KB1 and from a polychlorinated biphenyl contaminated site [58–60].

The paucity of high-quality bins reflects the diverse nature of the data; there are 2,588 genera in the combined assembly indicating that increased coverage is necessary to produce more MAGs, but the magnitude of the increase has yet to be determined. The detection of *Candidatus Omnitrophota* as one of the four high-quality bins demonstrates the power of WMS to detect new genomes yet to be cultivated. This group of nanobacteria is common in water and sediments and may be bacterial symbionts [61]. However, standards governing combining sequence data from multiple timepoints separated by years to produce MAGs are not yet available.

### Cometabolism and O_2_ production potential

Cometabolic pathways not linked to energy production occur in anaerobic and aerobic environments that coexistence at OH-contaminated sites [62] and NRAP is no exception. In addition to the detection of methane and methanogenic *Archaea* at NRAP (Fig 8A), genera with the potential for aerobic cometabolism also increase in abundance (Fig 8B). Methanotrophs *Methylocystis* and *Methylosinus* are good candidates for aerobic cometabolic functions (Fig 8B), but this remains circumstantial since epoxides are unstable and difficult to assay in the field. The production of chlorinated ethanes at an ERD site would indicate that aerobic cometabolism is occurring but ethanes were not assayed at NRAP. Although the increase in methane on-site can be attributed to methanogenic *Archaea*, the decrease in methane cannot be solely attributed to the action of methanotrophs since safety concerns required the installation of a vapor extraction system to collect and burn excess methane. *Xanthobacter* and *Dechloromonas* harbor *mo* genes, but only *Xanthobacter* is a literature-validated COMG.

The source of O_2_ in an anaerobic environment remains an enigma. Subsurface microenvironments, such as the soil-water interface, may sustain an O_2_ gradient. Alternatively, metabolic O_2_ may be available from microbial dismutation [31]. *Dechloromonas* is a facultative anaerobe known for its ability to degrade aromatic chlorinated solvents under anaerobic conditions and it can oxidize OH and produce O_2_ by chlorite dismutation. [31, 63]. The presence of *Dechloromonas* at NRAP is likely influenced by the presence of legacy petroleum hydrocarbon contamination in this aquifer. *Bradyrhizobium* is a diverse genus that includes nitrogen fixing members associated with legumes and has been studied from an agricultural perspective [64]. It is another candidate for dark oxygen production since it contains both *cld* and *nod* genes; it also contains *hdh* genes.

### The NRAP model for successful remediation

**Fig 12:**
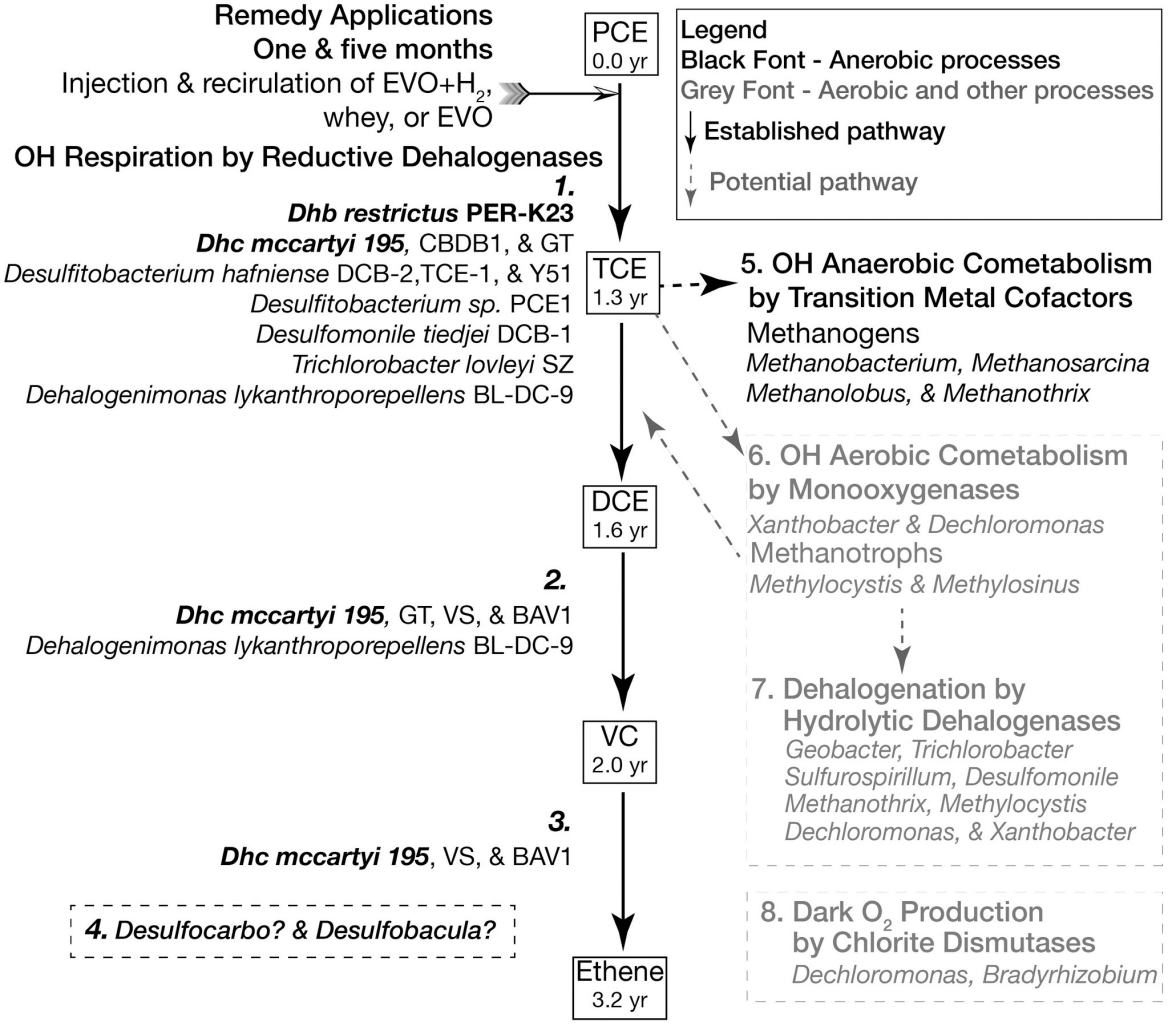
The NRAP model for successful ERD. The goal of ERD is the complete respiration of OHs and the presence of *Dhc*195 and *Dhb* (in bold) determined by qPCR in 2006 indicated that biostimulation was the remedy of choice. The numbers shown in the respiration pathway indicate when each compound reached its peak in the first 3.25 yrs. of the project. Relevant microbes whose presence is supported by MGT are indicated in the number lists. 1. PCE and TCE respiring strains 2. DCE respiring strains. 3. VC respiring strains. 4. Microbes that harbor *rdh* genes but are not validated as OHRG. TCE can also be subjected to anaerobic or aerobic cometabolic processes. 5. Methanogenic *Archaea*. 6. Methanotrophs and other *mo*-containing microbes can oxidase TCE, eliminating the double bond and producing unstable epoxides. 7. Hydrolytic dehalogenases are abundant in the NRAP microbiome, including in microbes that play other roles in dechlorination. The potential for products produced by aerobic cometabolism, such as chlorinated ethanes, remain to be verified. 8. The O_2_ for aerobic cometabolism may be produced by microbes that harbor chlorite dismutase.

The model in Fig 12 includes contributions from all data sources and highlights the importance of the community ecology perspective. WMS data is consistent with previous NRAP results, including qPCR [36]. WMS analysis reveals new taxa with the potential to directly contribute to the respiration of OH (*Desulfocarbo* and *Desulfobacula*). Other syntrophic interactions are supported, including cometabolism, hydrolytic dehalogenation, and dark oxygen production. Of interest from the community ecology and bioremediation perspectives is that individual microbes are capable of multiple roles in ERD. *Geobacter* is known to conduct electrons, produces an important class of cofactors, and has the potential for hydrolytic dehalogenation. *Dechloromonas* harbors genes that can facilitate aerobic cometabolism, hydrolytic dehalogenations, and dark O_2_ production.

### qPCR v. WMS v. 16S

This report validates that qPCR on baseline samples to detect taxa and genes capable of OH respiration is still an effective tool for remedy decisions. However, PCR-based techniques require an *a prior*i knowledge of the DNA sequences and do not facilitate the discovery of new taxa and genes that contribute to ERD. While WMS is a more costly alternative to MBTs that requires more DNA, it provides a more complete picture of the e-biome since lineage, coding regions, and abundance data can be extracted from the same dataset. Short read technology is currently the least expensive MGTs and its shortcomings are mitigated by alignment of short reads into longer scaffolds. Long-read technology for WMS is an alternative but it comes at a significantly increased cost. While 16S can replace methods such as DGGE and PFLA, it cannot provide information about genes and genomes not yet amplifiable with current primer sand amplification protocols. However, once the importance of new genes and genomes detected by WMS is established, new primers can be designed for qPCR methods that do not require DNA sequencing, such as microarrays. The WMS approach has the potential to identify solutions for stalled sites that mitigate the build-up of toxic by-products. A limitation of environmental metagenomics is the lack of standardization protocols, which makes it difficult to establish parameters such as minimum detection limit [65]. The development of control samples for environmental metagenomes will establish confidence in site-to-site comparisons. A significant advantage of MGTs is that DNA sequences can subjected to re-analysis as reference sequence databases are updated, resulting in new discoveries. This will be informative only if metadata are available, including detailed geochemical data and will require adherence to strict data management guidelines.

#### The future of NRAP

Due to the success of remediation in the shallow regions of the plume, most of the treatment infrastructure at NRAP was decommissioned. Once the manifolds and pumps were removed the use of submersible pumps was necessary for water sampling, which do not generate the pressure needed for filter capsules (Fig 1C), necessitating the development of a different on-site sampling system. Currently under testing is a battery powered system that uses vacuum instead of pressure and includes on-site sample processing that produces samples stable at ambient temperatures. This system has the added feature of estimating biomass on site (unpublished data). The deep section of the plume is still monitored once a year to determine contaminant levels and e-biomes were collected with the new system during the 2023 and 2024 monitoring events. Sequence data from these 16 and 17 year samples, the data from this analysis, and that from the 2016 paper [36] will be imported into the National Microbiome Data Collaborative database [66] and subjected to updated analyses using the most recent bioinformatics workflow and genome databases. The optimal design for a machine learning training set will inform how to best link the extensive site monitoring metadata available through the EPA with the sequence data.

## Conclusions

Metagenomics are a new class of techniques that are distinguished from MBTs by the use of NGS to generate data that can subjected to future analyses as databases and algorithms are updated. The NRAP results simultaneously highlights the potential of WMS results to formulate new treatment options for contaminated groundwater and the challenges associated with this technology. Among these challenges are the development of on-site sampling protocols that include guidelines relating biomass to sample volume of water necessary for adequate eDNA yield and the establishment of statistical procedures that include the minimum level of detection. At the sequencing level, the coverage needed for assembly of MAGs increases with sample richness, but guidelines have yet to be established. The bioinformatics challenges include read alignment algorithm refinement for low biomass/high diversity samples. Strict data management and availability guidelines are necessary to assure that sequence comparison to future versions of genomic databases are possible. A standardized metadata format is necessary to allow comparisons to other contaminated sites and ensure accurate training sets for machine learning.

## Supporting information

S1 File Supplemental text, S1–S3 Figs, S1–S9 Tables, and references. 16S rRNA Gene Amplification Protocol, Taxonomic Assignment Details, Lineage Correction, **S1 Table.** Lineage correction protocol, Visualization Details, **S2 Table**. Literature Validated OH Respiring Genera, **S3 Table.** Literature-Validated OH-Cometabolizing Genera, **S4 Table.** Phyla Limited to Baseline WMS-U, **S5 Table**. Other Candidatus Phyla in WMS-U, **S6 Table.** References for OH respiring strains detected at NRAP, **S7 Table.** Taxa Richness by Sample and by Method, **S8 Table**. *rdh* scaffold parameters, **S9 Table**. High-quality bins from NRAP combined assembly, **S2 Fig.** FIP-Selected Aerobic COMG, **S3 Fig.** Additional Genera with *hdh*-Containing Scaffolds at NRAP, **SI References**.
(DOCX)

S2 File NRAP data files. Raw and derived data in.xls format. **S2.0** Read Me tab contains abbreviations and explanations for the other three tabs. **S2.1** WMS-U Master Strain, **S2.2** 16S Master Genus, **S2.3** WMS-U + 16S by Genus.
(XLSX)

S3 File NRAP fig files. Data for figures in.xls format. **S3.0** Read Me tab contains abbreviations and explanations for the other 13 tabs. **S3.1** Fig 2 Overview, **S3.2** Fug 3 PCoA, **S3.3** Fig 4 Domain & CH_4_ Pies **S3.4** Fig 5 Phylum Cell Plot, **S3.5** Fig 5 Class cell plot **S3.6** Fig 6 FIP OHRG, **S3.7** Fig 7 Selected OHRG, **S3.8** Fig 8 OH respiration profiles, **S3.9** Fig 10 Selected COMG, **S3.10** Fig 11 Dark O2, **S3.11** S1 Fig FIP-selected COMG, **S3.12** S2 Fig FIP *hdh*.
(XLSX)

S4 File NRAP genes and genomes. Literature, database-derived, and other data in.xls format. **S4.0** Read Me tab contains abbreviations and explanations of the other nine tabs. **S4.1** Microbial Insights Results qPCR results **S4.2** Lit Val OHRG & COMG, **S4.3** IMG *rdh* genomes, **S4.4** IMG *mo* genomes, **S4.5** NRAP IMG *rdh* genus, **S4.6** IMG NRAP *mo* genus, **S4.7** NRAP *rdh* scaffolds, **S4.8** NRAP *dh* scaffolds, **S4.9** NRAP *hdh* by genus, **S4.10** Cl dismutases.
(XLSX)

S5 File PCoA interactive html file. This file will open a browser window, pointing at a marker opens a window that indicates the genus. Figure includes genera that have documented O_2_ requirements indicated by color, the domain is indicated by marker shape.
(HTML)
