# Supplementary material for: Whole metagenome sequencing and 16S rRNA gene amplicon analyses reveal the complex microbiome responsible for the success of enhanced in-situ reductive dechlorination (ERD) of a tetrachloroethene-contaminated Superfund site
Source: PLoS One. 2025 Feb 14;20(2):e0306503. doi: 10.1371/journal.pone.0306503 (PMC11828348; doi:10.1371/journal.pone.0306503)
Supplement: S1 File — 16S rRNA Gene Amplification Protocol, Taxonomic Assignment Details, Lineage Correction, S1 Table. Lineage correction protocol, Visualization Details, S2 Table. Literature Validated OH Respiring Genera, S3 Table. Literature-Validated OH-Cometabolizing Genera, S4 Table. Phyla Limited to Baseline WMS-U, S5 Table. Other Candidatus Phyla in WMS-U, S6 Table. References for OH respiring strains detected at NRAP, S7 Table. Taxa Richness by Sample and by Method, S8 Table. rdh scaffold parameters, S9 Table. High-quality bins from NRAP combined assembly, S2 Fig. FIP-Selected Aerobic COMG, S3 Fig. Additional Genera with hdh-Containing Scaffolds at NRAP, SI References. (DOCX) [file pone.0306503.s001.docx]

Supplemental File 1

Whole metagenome sequencing and 16S rRNA gene amplicon analyses reveal the complex microbiome responsible for the success of enhanced in-situ reductive dechlorination (ERD) of a tetrachloroethene-contaminated Superfund site

Short Title: Metagenomic profiles of aquifer microbes responding to bioremediation of organohalide contamination

Rebecca A. Reiss^1,2*¶^, Peter A. Guerra^3¶^, Oleg Makhnin^4&^, Matthew Kellom^5&^

^1^Biology Department, New Mexico Tech, Socorro New Mexico, United States of America

^2^LifeScience Testing and Analysis, Albuquerque, NM, United States of America

^3^Lynker Corporation, Albuquerque, New Mexico, United States of America

^4^Mathematics Department, New Mexico Tech, Socorro New Mexico, United States of America

^5^Department of Energy Joint Genome Institute, Lawrence Berkeley National Laboratory, Berkeley, California, United States of America

*Corresponding author

E-mail: [rebecca.reiss@nmt.edu](mailto:rebecca.reiss@nmt.edu)

[16S rRNA Gene Amplicon Bioinformatics Workflow 2](#_Toc183586663)

[Taxonomic Assignment Details 2](#_Toc183586664)

[Lineage Correction 3](#_Toc183586665)

[Table S1. Lineage Correction Protocol 3](#_Toc183586666)

[Visualization details 4](#_Toc183586667)

[Table S2. Literature Validated OH Respiring Genera 5](#_Toc183586668)

[Table S3. Literature-Validated OH- Cometabolic Genera 6](#_Toc183586669)

[Table S4. Phyla Limited to Baseline WMS-U 7](#_Toc183586670)

[Table S5. Other *Candidatus* Phyla in WMS-U 7](#_Toc183586671)

[Table S6. References for OH Respiring Strains Detected at NRAP 8](#_Toc183586672)

[Table S7. Taxa Richness by Sample and by Method 8](#_Toc183586673)

[Table S8. *rdh* Scaffold Parameters. 9](#_Toc183586674)

[Table S9. High-Quality Bins from NRAP Combined Assembly 10](#_Toc183586675)

[Fig. S1. FIP-Selected Aerobic COMG. 11](#_Toc183586676)

[Fig. S2. Additional Genera with *hdh*-Containing Scaffolds at NRAP. 12](#_Toc183586677)

[S1 References 13](#_Toc183586678)

16S rRNA Gene Amplicon Bioinformatics Workflow

The 16SrRNA sequences were quality and contamination filtered with FLASH (v1.2.6) and duk (v1.05), respectively. [1, 2] Reads were pair-merged into amplicons with FLASH, then clustered into operational taxonomic units with USEARCH (v7.0.959 i86linux32) [3], checked for chimeras with UCHIME [4], and annotated with the Ribosomal Database Project (RDP) Classifier (v2.5) [5].

Taxonomic Assignment Details

Unassembled 150 bp reads are translated into six reading frames of 50 amino acids and is assigned a strain-level taxonomic identification (IDed) when at least 30% of the amino acids of a reading frame match a genome from the IMG/M reference database and at least 70% of the gene sequence is covered by the alignment, otherwise the read remains unidentified.

Filtered reads from each of the six WGS metagenomes were assembled individually and were also combined into one assembly and annotated through GMAP. Assembly involves first aligning reads into overlapping contiguous reads (contigs), with or without ambiguous bases. Protein coding regions of scaffolds can then be predicted based on gene sequence models and subsequently annotated according to reference databases of protein sequences. Read mapping to scaffolds produces an overall length and the number of times each base is covered by a read averaged over all bases, the depth. Scaffold size is the length times the depth as measured in bp.

Scaffold taxonomic assignments are made when the percentage of predicted protein-coding regions in a scaffold exceeds 50% for any taxonomic level. For example, if at least 50% of the coding sequences match the designated species of a reference genome, then the species level assignment is made. However, if species match does not exceed 50%, then the genus-level assignment was made if at least 50% of the coding sequences matched a genus. This approach results in a variety of taxonomic level assignments for scaffolds, from no assignment to the genome-level assignments. This strategy takes advantage of the additional information provided by assembly that is not available in MSS-U, in which a taxonomic assignment is made only if the match to a reference isolate genome is above 70% amino acid identity.

Lineage Correction

This process is based on the structure of taxonomic lineages, which is an ontology where any level can have multiple children, but only one parent. For example, all members of the genus Bacillus should have the same lineage. Changes in nomenclature at the higher levels can result in different lineages, for Bacillus the validly published and correct order name is Caryophanalesbut the validly published synonym, Bacillales, is still used in some databases, especially for older entries. The process starts with tabulating the file on the entire lineage and then the resulting file this tabulated on a level (e.g., genus). This produces a list of the number of lineages for each genus, which should be one. Genera with multiple lineages were selected and the lineage corrected based upon the most recent nomenclature.

Table S1. Lineage Correction Protocol

| Tabulate | Tabulate on all levels above the one in question. For example, to make sure that all genera have only one path, it is necessary to include phylum, class, order, and family in the tabulation. |
| --- | --- |
| New data table | Make into a data table. |
| Tabulate | Tabulate on the level in questions (genus for example). |
| New data table | Make into a data table and select any genus for which N > 1. |
| Select | Go to original data table, select the members of taxa in question and create a linked subset. |
| Look-up in LPSN | Consult LPSN to find the correct name. Often the problem is that older entries in the database may use nomenclature that has been changed. For example, the order *Clostridiales* is now called *Eubacteriales*, so both names made be in the data depending upon when the reference strain was entered. Make the necessary changes. |
| Tabulate | Once complete, there will be only one lineage for each OTU. |

Visualization details

Cell plots were generated in JMP after applying binning formulas for each RPM. A dummy variable for each bin was added to ensure that all columns were uniformly scaled, these variables are shown in supplemental files (S3 File) but are hidden in the figures. Line graphs of specific genera were generated by selecting the genus in the file containing WMS and 16S, creating a stacked file, and using the Graph Builder function in JMP to overlay the 16S rRNA and WMS-U data for the SAE timepoints.

Table S2. Literature Validated OH Respiring Genera

| Domain | Phylum | Genus [ref] |
| --- | --- | --- |
| *Bacteria* | *Bacillota* | *Acetobacterium* [6] |
| *Bacteria* | *Acidobacteriota* | *Acidobacterium* [6] |
| *Bacteria* | *Pseudomonadota* | *Ahrensia* [7] |
| *Bacteria* | *Pseudomonadota* | *Anaeromyxobacter* [8] |
| *Bacteria* | *Bacillota* | *Clostridium* [6] |
| *Bacteria* | *Bacillota* | *Dehalobacter* [9] |
| *Bacteria* | *Chloroflexota* | *Dehalococcoides* [6] |
| *Bacteria* | *Chloroflexota* | *Dehalogenimonas* [10] |
| *Bacteria* | *Bacillota* | *Desulfitobacterium* [11] |
| *Bacteria* | *Pseudomonadota* | *Desulfoluna* [8] |
| *Bacteria* | *Pseudomonadota* | *Desulfomonile* [8] |
| *Bacteria* | *Pseudomonadota* | *Desulfovibrio* [8] |
| *Bacteria* | *Pseudomonadota* | *Desulfuromonas [8]* |
| *Bacteria* | *Bacillota* | *Dethiobacter* [7] |
| *Bacteria* | *Pseudomonadota* | *Enterobacter* [6] |
| *Archaea* | *Methanobacteriota* | *Ferroglobus* [7] |
| *Bacteria* | *Pseudomonadota* | *Geobacter* [8] |
| *Bacteria* | *Bacillota* | *Heliobacterium* [7] |
| *Bacteria* | *Pseudomonadota* | *Jannaschia* [12] |
| *Archaea* | *Methanobacteriota* | *Methanobacterium* [6] |
| *Archaea* | *Methanobacteriota* | *Methanolobus* [6] |
| *Archaea* | *Methanobacteriota* | *Methanosarcina* [6] |
| *Bacteria* | *Pseudomonadota* | *Photobacterium* [13] |
| *Bacteria* | *Actinomycetota* | *Propionibacterium* [6] |
| *Bacteria* | *Pseudomonadota* | *Ruegeria* [7] |
| *Bacteria* | *Pseudomonadota* | *Shewanella* [8] |
| *Bacteria* | *Bacillota* | *Sporomusa* [14] |
| *Bacteria* | *Pseudomonadota* | *Sulfurospirillum* [15] |
| *Bacteria* | *Thermotogota* | *Thermotoga* [7] |
| *Bacteria* | *Pseudomonadota* | *Vibrio* [7] |

Table S3. Literature-Validated OH- Cometabolic Genera

| Domain | Phylum | Genus [ref] |
| --- | --- | --- |
| *Bacteria* | *Pseudomonadota* | *Agrobacterium* [16] |
| *Bacteria* | *Pseudomonadota* | *Alcaligenes* [6] |
| *Bacteria* | *Pseudomonadota* | *Ancylobacter* [16] |
| *Bacteria* | *Actinomycetota* | *Arthrobacter* [16] |
| *Bacteria* | *Pseudomonadota* | *Azotobacter* [16] |
| *Bacteria* | *Bacillota* | *Bacillus* [6] |
| *Bacteria* | *Pseudomonadota* | *Bordetella* [6] |
| *Bacteria* | *Pseudomonadota* | *Brevundimonas* [6] |
| *Bacteria* | *Pseudomonadota* | *Burkholderia* [6] |
| *Bacteria* | *Pseudomonadota* | *Comamonas* [6] |
| *Bacteria* | *Chloroflexota* | *Dehalobium* [17] |
| *Bacteria* | *Pseudomonadota* | *Klebsiella* [18] |
| *Bacteria* | *Bacillota* | *Lysinibacillus* [16] |
| *Bacteria* | *Pseudomonadota* | *Mesorhizobium* [16] |
| *Bacteria* | *Pseudomonadota* | *Methylobacter* [6] |
| *Bacteria* | *Pseudomonadota* | *Methylobacterium* [16] |
| *Bacteria* | *Pseudomonadota* | *Methylococcus* [6] |
| *Bacteria* | *Pseudomonadota* | *Methylocystis* [6] |
| *Bacteria* | *Pseudomonadota* | *Methylomonas* [6] |
| *Bacteria* | *Pseudomonadota* | *Methylosinus* [6] |
| *Bacteria* | *Pseudomonadota* | *Moraxella* [16] |
| *Bacteria* | *Actinomycetota* | *Mycobacterium* [6] |
| *Bacteria* | *Pseudomonadota* | *Nitrosomonas* [6] |
| *Bacteria* | *Actinomycetota* | *Nocardioides* [6] |
| *Bacteria* | *Pseudomonadota* | *Ochrobactrum* [6] |
| *Bacteria* | *Pseudomonadota* | *Paracoccus* [16] |
| *Bacteria* | *Pseudomonadota* | *Polaromonas* [6] |
| *Bacteria* | *Pseudomonadota* | *Pseudoalteromonas* [16] |
| *Bacteria* | *Pseudomonadota* | *Pseudomonas* [6] |
| *Bacteria* | *Pseudomonadota* | *Psychromonas* [16] |
| *Archaea* | *Methanobacteriota* | *Pyrococcus* [16] |
| *Bacteria* | *Pseudomonadota* | *Ralstonia* [6] |
| *Bacteria* | *Pseudomonadota* | *Rhizobium* [16] |
| *Bacteria* | *Actinomycetota* | *Rhodococcus* [6] |
| *Bacteria* | *Pseudomonadota* | *Rhodoferax* [6] |
| *Bacteria* | *Pseudomonadota* | *Serratia* [16] |
| *Bacteria* | *Pseudomonadota* | *Sphingopyxis* [19] |
| *Bacteria* | *Pseudomonadota* | *Stenotrophomonas* [20] |
| *Archaea* | *Thermoproteota* | *Sulfolobus* [21] |
| *Bacteria* | *Bacillota* | *Tissierella* [6] |
| *Bacteria* | *Pseudomonadota* | *Variovorax* [6] |
| *Bacteria* | *Pseudomonadota* | *Xanthobacter* [6] |

Table S4. Phyla Limited to Baseline WMS-U

| Domain | Phylum (a.k.a) |
| --- | --- |
| *Archaea* | *Candidatus Aigarchaeota* [22] |
| *Bacteria* | *Armatimonadota (OP10)* |
| *Bacteria* | *Atribacterota (Atribacteria)* |
| *Bacteria* | *Candidatus Aerophobetes* [23] |
| *Bacteria* | *Candidatus Aminicenantes* [23] |
| *Bacteria* | *Candidatus Calescamantes* [23] |
| *Bacteria* | *Candidatus Diapherotrites* [23] |
| *Bacteria* | *Candidatus Gracilibacteria* [23] |
| *Bacteria* | *Candidatus Hydrogenedentes* [23] |
| *Bacteria* | *Candidatus Latescibacteria* [23] |
| *Bacteria* | *Candidatus Omnitrophica* [23] |
| *Bacteria* | *Candidatus Parcubacteria* [23] |
| *Bacteria* | *Candidatus Sumerlaeota* [24] |

Table S5. Other *Candidatus* Phyla in WMS-U

| Domain | Phylum |
| --- | --- |
| *Bacteria* | *Candidatus Absconditabacteria* [25] |
| *Bacteria* | *Candidatus Cloacimonadota* [26] |
| *Archaea* | *Candidatus Geoarchaeota* [27] |
| *Archaea* | *Candidatus Korarchaeota* [28] |
| *Bacteria* | *Candidatus Marinimicrobia* [26] |
| *Bacteria* | *Candidatus Microgenomates* [26] |
| *Bacteria* | *Candidatus Poribacteria* [29] |
| *Bacteria* | *Candidatus Saccharibacteria* [30] |
| *Archaea* | *Candidatus Thermoplasmatota* [26]­ |

Table S6. References for OH Respiring Strains Detected at NRAP

| Strain | Reference |
| --- | --- |
| *Dehalobacter restrictus PER-K23* | [6] |
| *Dehalococcoides mccartyi CBDB1* | [6] |
| *Desulfitobacterium hafniense DCB-2* | [6] |
| *Desulfitobacterium hafniense TCE-1* | [31] |
| *Desulfitobacterium sp. PCE1, DSM 10344* | [6] |
| *Desulfitobacterium hafniense Y51* | [6] |
| *Desulfomonile tiedjei DCB-1, DSM 6799* | [6] |
| *Sulfurospirillum multivorans DSM 12446** | [32] |
| *Trichlorobacter lovleyi SZ* | [6] |
| *Dehalogenimonas lykanthroporepellens BL-DC-9* | [33] |
| *Dehalococcoides mccartyi DCMB5* | [6] |
| *Dehalococcoides mccartyi GT* | [34] |
| *Dehalococcoides mccartyi BTF08* | [6] |
| *Dehalococcoides mccartyi 195* | [6] |
| *Dehalococcoides mccartyi VS* | [34] |
| *Dehalococcoides mccartyi BAV1* | [6] |

Table S7. Taxa Richness by Sample and by Method

|  | Phylum | | | Class | | | Order | | | Family | | | Genus | | |
| --- | --- | --- | --- | --- | --- | --- | --- | --- | --- | --- | --- | --- | --- | --- | --- |
| Method | 16S^1^ | WU^2^ | WA^3^ | 16S | WU | WA | 16S | WU | WA | 16S | WU | WA | 16S | WU | WA |
| Sample |  |  |  |  |  |  |  |  |  |  |  |  |  |  |  |
| Overall | 19 | 57 | 50 | 38 | 119 | 122 | 69 | 224 | 257 | 127 | 460 | 563 | 241 | 1499 | 2557 |
| SAE3-00 | 18 | 51 |  | 33 | 106 |  | 58 | 205 |  | 98 | 428 |  | 159 | 1358 |  |
| HSE6-00^4^ | 15 |  |  | 28 |  |  | 60 |  |  | 106 |  |  | 171 |  |  |
| SAE3-04 | 18 | 44 |  | 30 | 94 |  | 54 | 183 |  | 84 | 373 |  | 136 | 1141 |  |
| HSE6-04 | 17 | 44 |  | 32 | 94 |  | 53 | 183 |  | 83 | 373 |  | 134 | 1142 |  |
| SAE3-23 | 18 | 44 |  | 33 | 94 |  | 58 | 183 |  | 95 | 373 |  | 157 | 1143 |  |
| HSE6-23 | 17 | 44 |  | 35 | 94 |  | 58 | 182 |  | 91 | 372 |  | 145 | 1141 |  |
| SAE3-39 | 18 | 44 |  | 33 | 94 |  | 54 | 183 |  | 103 | 373 |  | 180 | 1143 |  |

1. 16S rRNA gene amplicon

2. WMS-U

3. WMS-A

4. Based on a single replicate of 16S rRNA gene amplification, no WMS data are available.

Table S8. *rdh* Scaffold Parameters.

Scaffolds in bold are shown in Fig. 9

| Scaffold OID | *rdh* protein Family Designation | Taxonomic identification | Length (bp) | Depth^1^ | # of Genes |
| --- | --- | --- | --- | --- | --- |
| Ga0272380_10027961 | pfam13486^2^ | *Dehalobacter* sp. FTH1 | 5689 | 23 | 6 |
| Ga0272380_10029723 | pfam13486 | *Dehalococcoides mccartyi* 195 | 5431 | 15 | 7 |
| Ga0272380_10045439 | pfam13486 | *Dehalococcoides mccartyi* 11a5 | 3940 | 13 | 5 |
|  | COG 1600^3^ |  |  |  |  |
| Ga0272380_10049508 | pfam10518^4^ | *Dehalococcoides mccartyi* MB | 3700 | 13 | 6 |
|  | COG 1600 |  |  |  |  |
| Ga0272380_10057639 | pfam13486 | *Dehalococcoides mccartyi* 195 | 3321 | 14 | 4 |
| Ga0272380_10104487 | pfam13486 | *Dehalococcoides mccartyi* CG4 | 2218 | 12 | 4 |
| Ga0272380_10183744 | pfam13484^5^ | *Dehalococcoides mccartyi* 195 | 1536 | 15 | 2 |
| Ga0272380_10251625 | pfam10518 | *Dehalococcoides* mccartyi CG4 | 1256 | 11 | 2 |
| Ga0272380_10624771 | pfam13486 | *Dehalococcoides* *mccartyi* MB | 699 | 5 | 1 |
| Ga0272380_10740354 | pfam13486 | *Desulfocarbo indianensis* SCBM | 625 | 5 | 1 |
| Ga0272380_10843876 | pfam13486 | *Dehalogenimonas* sp. WBC-2 | 573 | 4 | 1 |
| Ga0272380_11007108 | pfam13486 | *Dehalococcoides mccartyi* MB | 508 | 15 | 1 |
| Ga0272380_11530855 | pfam13486 | *Desulfobacula phenolica* DSM 3384 | 385 | 5 | 1 |
| Ga0272380_11723655 | pfam13486 | *Dehalobacter* sp. CF | 357 | 3 | 1 |
| Ga0272380_11763765 | pfam13486 | *Dehalobacter* sp. UNSWDHB | 351 | 5 | 1 |
| Ga0272380_11871684 | pfam13486 | *Dehalobacter* sp. FTH1 | 338 | 4 | 1 |
| Ga0272380_12175589 | pfam13486 | *Dehalobacter restrictus* DSM 9455 | 307 | 4 | 1 |
| Ga0272380_12426655 | pfam13486 | *Dehalococcoides mccartyi* CG4 | 287 | 11 | 1 |
| Ga0272380_12465994 | pfam13486 | *Dehalococcoides mccartyi* DCMB5 | 285 | 3 | 1 |
| Ga0272380_12569262 | pfam13486 | not identified | 278 | 4 | 1 |

1. Average number of reads per base

2. Protein Familiy (pfam) reductive dehalogenase subunit-like protein

3. Clusters of Orthologous Gene (COG) epoxyqueuosine reductase QueG

4. reductive dehalogenase subunit-like protein/TAT (twin-arginine translocation) pathway-exported protein

5. reductive dehalogenase subunit-like protein/4Fe-4S dicluster protein

Table S9. High-Quality Bins from NRAP Combined Assembly

| Bin ID* | Taxon | Bin Completeness | Contamination | 5S rRNA | 16S rRNA | 23S rRNA | tRNA Genes | Gene Count | Scaffold Count | Total # Bases |
| --- | --- | --- | --- | --- | --- | --- | --- | --- | --- | --- |
| 3300005323_12 | *Archaea; Methanoregulaceae* | 93.75 | 0.66 | 1 | 1 | 1 | 46 | 2761 | 61 | 2634707 |
| 3300005323_13 | *Archaea; Methanobacterium formicicum* | 93.47 | 0.8 | 3 | 1 | 2 | 44 | 2706 | 39 | 2584797 |
| 3300005323_14 | *Bacteria; Sulfuricurvum kujiense* | 93.03 | 1.43 | 1 | 1 | 1 | 43 | 2596 | 199 | 2356281 |
| 3300005323_16 | *Bacteria; Omnitrophota;* | 91.76 | 3.76 | 1 | 1 | 1 | 45 | 1784 | 3 | 1935083 |

*Bin ID can be used in JGI data portal to find bin data file


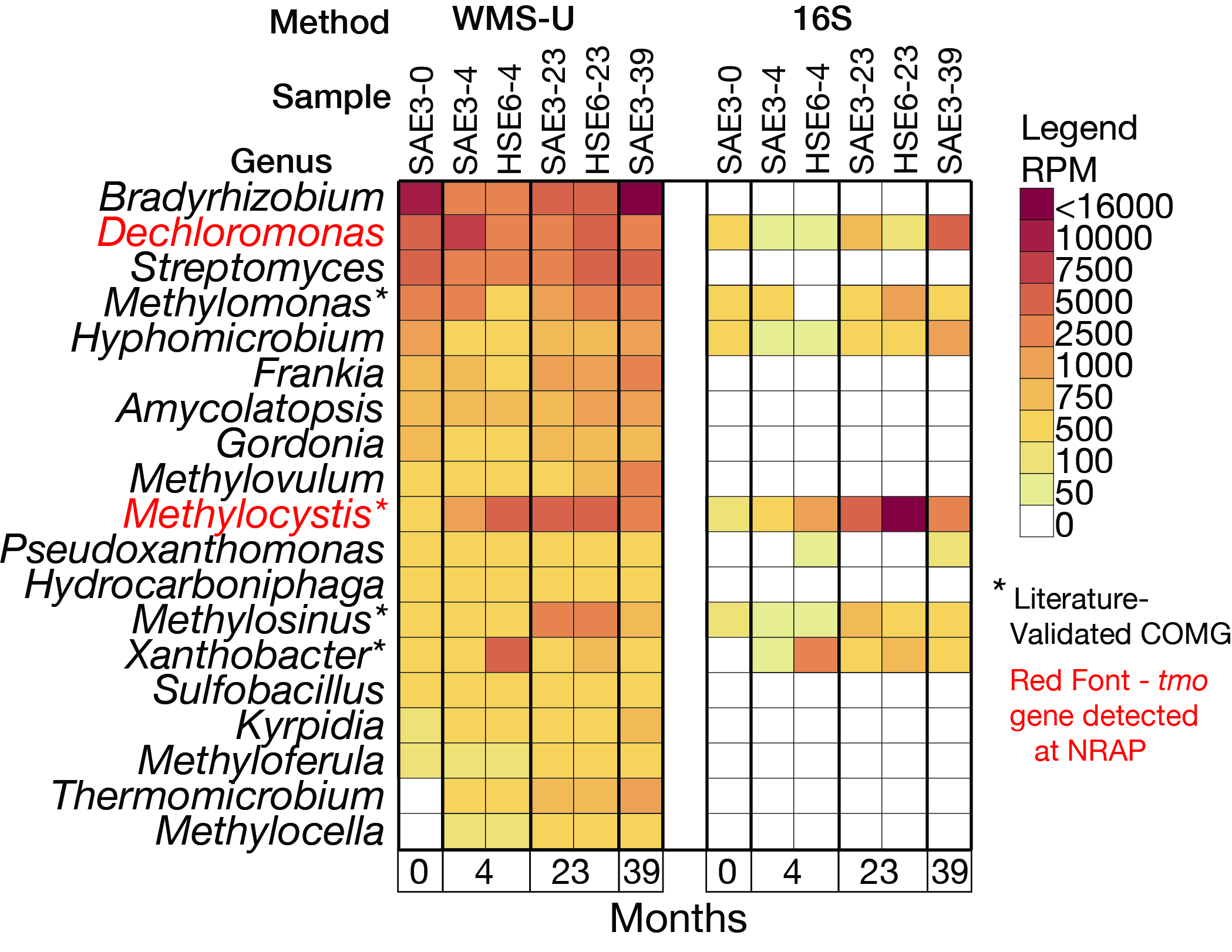


Fig. S1. FIP-Selected Aerobic COMG.

These genera include IMG/M genomes with either *mmo* or *tmo* genes that increase in abundance at NRAP for at least one timepoint.


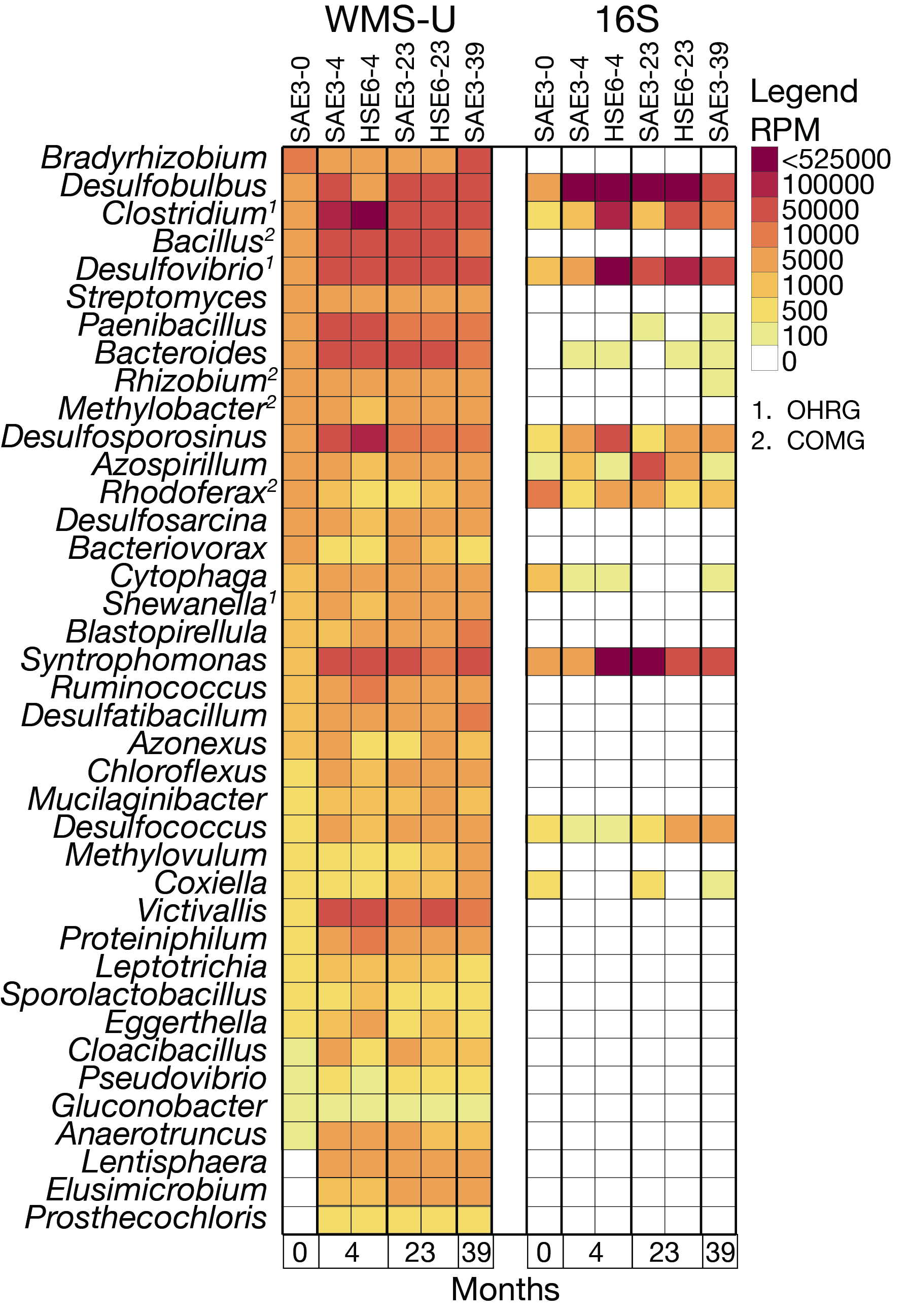


Fig. S2. Additional Genera with *hdh*-Containing Scaffolds at NRAP.

The list excludes eight previously described genera. Superscripts indicate literature-validated OHRG or COMG.

S1 References

1. Magoc T, Salzberg SL. FLASH: fast length adjustment of short reads to improve genome assemblies. Bioinformatics. 2011;27(21):2957-63. doi: 10.1093/bioinformatics/btr507

2. Li M, Copeland A, Han J. DUK - A fast and efficient kmer based sequence matching tool. Lawrence Berkeley National Laboratory: Lawrence Berkeley National Laboratory; 2011 2011. Report No.: LBNL Report #: LBNL-4516E-Poster Contract No.: LBNL Report #: LBNL-4516E-Poster. Available from: <https://escholarship.org/uc/item/8pf7407j>

3. Edgar RC. Search and clustering orders of magnitude faster than BLAST. Bioinformatics. 2010;26(19):2460-1. doi: 10.1093/bioinformatics/btq461

4. Edgar RC, Haas BJ, Clemente JC, Quince C, Knight R. UCHIME improves sensitivity and speed of chimera detection. Bioinformatics. 2011;27(16):2194-200. doi: 10.1093/bioinformatics/btr381

5. Wang Q, Garrity GM, Tiedje JM, Cole JR. Naive Bayesian classifier for rapid assignment of rRNA sequences into the new bacterial taxonomy. Appl Environ Microbiol. 2007;73(16):5261-7. doi: 10.1128/aem.00062-07

6. Dolinova I, Strojsova M, Cernik M, Nemecek J, Machackova J, Sevcu A. Microbial degradation of chloroethenes: a review. Environ Sci Pollut Res Int. 2017;24(15):13262-83. doi: 10.1007/s11356-017-8867-y

7. Hug LA, Maphosa F, Leys D, Loffler FE, Smidt H, Edwards EA, et al. Overview of organohalide-respiring bacteria and a proposal for a classification system for reductive dehalogenases. Philos Trans R Soc Lond B Biol Sci. 2013;368(1616):20120322. doi: 10.1098/rstb.2012.0322

8. Atashgahi S, Lu Y, Smidt H. Overview of known organohalide-respiring bacteria—phylogenetic diversity and environmental distribution. In: Adrian L, Löffler FE, editors. Organohalide-Respiring Bacteria. Berlin, Heidelberg: Springer Berlin Heidelberg; 2016. p. 63-105. doi: 10.1007/978-3-662-49875-0_5.

9. Maillard J, Holliger C. The genus *Dehalobacter*. In: Adrian L, Löffler FE, editors. Organohalide-Respiring Bacteria2016. p. 153-71. doi: 10.1007/978-3-662-49875-0_8.

10. Moe WM, Rainey FA, Yan J. The Genus *Dehalogenimonas*. In: Adrian L, Löffler FE, editors. Organohalide-Respiring Bacteria. Berlin, Heidelberg: Springer Berlin Heidelberg; 2016. p. 137-51. doi: 10.1007/978-3-662-49875-0_7.

11. Futagami T, Furukawa K. The genus *Desulfitobacterium*. In: Adrian L, Löffler FE, editors. Organohalide-Respiring Bacteria. Berlin, Heidelberg: Springer Berlin Heidelberg; 2016. p. 173-207. doi: 10.1007/978-3-662-49875-0_9.

12. Chan WY, Wong M, Guthrie J, Savchenko AV, Yakunin AF, Pai EF, et al. Sequence- and activity-based screening of microbial genomes for novel dehalogenases. Microb Biotechnol. 2010;3(1):107-20. doi: 10.1111/j.1751-7915.2009.00155.x

13. Buttet GF, Willemin MS, Hamelin R, Rupakula A, Maillard J. The membrane-bound C subunit of reductive dehalogenases: Topology analysis and reconstitution of the fmn-binding domain of PceC. Frontiers in Microbiology. 2018;9(775). doi: 10.3389/fmicb.2018.00755

14. Terzenbach DP, Blaut M. Transformation of tetrachloroethylene to trichloroethylene by homoacetogenic bacteria. FEMS Microbiology Letters. 1994;123(1-2):213-8. doi: 10.1111/j.1574-6968.1994.tb07224.x

15. Buttet GF, Murray AM, Goris T, Burion M, Jin B, Rolle M, et al. Coexistence of two distinct *Sulfurospirillum* populations respiring tetrachloroethene—genomic and kinetic considerations. FEMS Microbiology Ecology. 2018;94(5):fiy018. doi: 10.1093/femsec/fiy018

16. Wang Y, Xiang Q, Zhou Q, Xu J, Pei D. Mini Review: Advances in 2-Haloacid Dehalogenases. Frontiers in Microbiology. 2021;12. doi: 10.3389/fmicb.2021.758886

17. Kittelmann S, Friedrich MW. Novel uncultured *Chloroflexi* dechlorinate perchloroethene to trans-dichloroethene in tidal flat sediments. Environmental Microbiology. 2008;10(6):1557-70. doi: 10.1111/j.1462-2920.2008.01571.x

18. Tahya C, Ratnaningsih E. Cloning and sequencing of haloacid dehalogenase gene from *Klebsiella pneumoniae ITB1*. Procedia Chemistry. 2015;16:121-8. doi:

19. Varzaghani NB, Shokrollahzadeh S, Farazmand A. Biodegradation of tetrachloroethylene by a newly isolated aerobic *Sphingopyxis ummariensis VR13*. Korean Journal of Chemical Engineering. 2019;36(8):1305-12. doi: 10.1007/s11814-019-0303-1

20. Mukherjee P, Roy P. Persistent organic pollutants induced protein expression and immunocrossreactivity by *Stenotrophomonas maltophilia PM102*: a prospective bioremediating candidate. Biomed Res Int. 2013;2013:714232. doi: 10.1155/2013/714232

21. Bachas-Daunert PG, Law SA, Wei Y. Characterization of a recombinant thermostable dehalogenase isolated from the hot spring thermophile *Sulfolobus tokodaii*. Applied Biochemistry and Biotechnology. 2009;159(2):382-93. doi: 10.1007/s12010-009-8589-9

22. Beam JP, Jay ZJ, Schmid MC, Rusch DB, Romine MF, Jennings Rde M, et al. Ecophysiology of an uncultivated lineage of *Aigarchaeota* from an oxic, hot spring filamentous 'streamer' community. ISME J. 2016;10(1):210-24. doi: 10.1038/ismej.2015.83

23. Rinke C, Schwientek P, Sczyrba A, Ivanova NN, Anderson IJ, Cheng JF, et al. Insights into the phylogeny and coding potential of microbial dark matter. Nature. 2013;499(7459):431-7. doi: 10.1038/nature12352

24. Kadnikov VV, Mardanov AV, Beletsky AV, Rakitin AL, Frank YA, Karnachuk OV, et al. Phylogeny and physiology of candidate phylum BRC1 inferred from the first complete metagenome-assembled genome obtained from deep subsurface aquifer. Syst Appl Microbiol. 2019;42(1):67-76. doi: 10.1016/j.syapm.2018.08.013

25. Hug LA, Baker BJ, Anantharaman K, Brown CT, Probst AJ, Castelle CJ, et al. A new view of the tree of life. Nat Microbiol. 2016;1:16048. doi: 10.1038/nmicrobiol.2016.48

26. Rinke C, Rubino F, Messer LF, Youssef N, Parks DH, Chuvochina M, et al. A phylogenomic and ecological analysis of the globally abundant Marine Group II archaea (Ca. *Poseidoniales* ord. nov.). The ISME Journal. 2019;13(3):663-75. doi: 10.1038/s41396-018-0282-y

27. Kozubal MA, Romine M, Jennings R, Jay ZJ, Tringe SG, Rusch DB, et al. *Geoarchaeota*: a new candidate phylum in the Archaea from high-temperature acidic iron mats in Yellowstone National Park. ISME J. 2013;7(3):622-34. doi: 10.1038/ismej.2012.132

28. Ludwig W, Klenk H-P. Overview: A Phylogenetic Backbone and Taxonomic Framework for Procaryotic Systematics. In: Boone DR, Castenholz RW, Garrity GM, editors. Bergey’s Manual® of Systematic Bacteriology: Volume One : The Archaea and the Deeply Branching and Phototrophic Bacteria. New York, NY: Springer New York; 2001. p. 49-65. doi: 10.1007/978-0-387-21609-6_8.

29. Fieseler L, Horn M, Wagner M, Hentschel U. Discovery of the novel candidate phylum "*Poribacteria*" in marine sponges. Appl Environ Microbiol. 2004;70(6):3724-32. doi: 10.1128/AEM.70.6.3724-3732.2004

30. Albertsen M, Hugenholtz P, Skarshewski A, Nielsen KL, Tyson GW, Nielsen PH. Genome sequences of rare, uncultured bacteria obtained by differential coverage binning of multiple metagenomes. Nat Biotechnol. 2013;31(6):533-8. doi: 10.1038/nbt.2579

31. Duret A, Holliger C, Maillard J. The physiological opportunism of *Desulfitobacterium hafniense* strain TCE1 towards organohalide respiration with tetrachloroethene. Appl Environ Microbiol. 2012;78(17):6121-7. doi: 10.1128/aem.01221-12

32. Goris T, Schubert T, Gadkari J, Wubet T, Tarkka M, Buscot F, et al. Insights into organohalide respiration and the versatile catabolism of *Sulfurospirillum multivorans* gained from comparative genomics and physiological studies. Environ Microbiol. 2014;16(11):3562-80. doi: 10.1111/1462-2920.12589

33. Moe WM, Yan J, Nobre MF, da Costa MS, Rainey FA. *Dehalogenimonas lykanthroporepellens* gen. nov., sp. nov., a reductively dehalogenating bacterium isolated from chlorinated solvent-contaminated groundwater. International Journal of Systematic and Evolutionary Microbiology. 2009;59(11):2692-7. doi: <https://doi.org/10.1099/ijs.0.011502-0>

34. Loffler FE, Yan J, Ritalahti KM, Adrian L, Edwards EA, Konstantinidis KT, et al. *Dehalococcoides mccartyi* gen. nov., sp. nov., obligately organohalide-respiring anaerobic bacteria relevant to halogen cycling and bioremediation, belong to a novel bacterial class, *Dehalococcoidia* classis nov., order *Dehalococcoidales* ord. nov. and family *Dehalococcoidaceae* fam. nov., within the phylum *Chloroflexi*. Int J Syst Evol Microbiol. 2013;63(Pt 2):625-35. doi: 10.1099/ijs.0.034926-0
