# Supplementary material for: Whole metagenome sequencing and 16S rRNA gene amplicon analyses reveal the complex microbiome responsible for the success of enhanced in-situ reductive dechlorination (ERD) of a tetrachloroethene-contaminated Superfund site
Source: PLoS One. 2025 Feb 14;20(2):e0306503. doi: 10.1371/journal.pone.0306503 (PMC11828348; doi:10.1371/journal.pone.0306503)
Supplement: S5 File — This file will open a browser window, pointing at a marker opens a window that indicates the genus. Figure includes genera that have documented O2 requirements indicated by color, the domain is indicated by marker shape. (HTML) [file pone.0306503.s005.html]

NRAP PCoA\_Genus 11.24.24 2 - Graph Builder 4
